# Agronomic strategies to enhance the early vigor and yield of maize. Part I: the role of seed applied biostimulant, hybrid and starter fertilization on rhizosphere bacteria profile and diversity

**DOI:** 10.3389/fpls.2023.1240310

**Published:** 2023-11-02

**Authors:** Gergely Ujvári, Luca Capo, Arianna Grassi, Caterina Cristani, Irene Pagliarani, Alessandra Turrini, Massimo Blandino, Manuela Giovannetti, Monica Agnolucci

**Affiliations:** ^1^ Department of Agriculture, Food and Environment, University of Pisa, Pisa, Italy; ^2^ Department of Agriculture, Forest and Food Sciences, University of Turin, Grugliasco, Italy

**Keywords:** maize, seed treatment, biostimulant, rhizosphere, bacterial communities, plant growth promoting bacteria, *Bacillus amyloliquefaciens*, diammonium phosphate

## Abstract

The sustainable intensification of maize-based systems may reduce greenhouse-gas emissions and the excessive use of non-renewable inputs. Considering the key role that the microbiological fertility has on crop growth and resilience, it is worth of interest studying the role of cropping system on the rhizosphere bacterial communities, that affect soil health and biological soil fertility. In this work we monitored and characterized the diversity and composition of native rhizosphere bacterial communities during the early growth phases of two maize genotypes of different early vigor, using a nitrogen (N)-phosphorus (P) starter fertilization and a biostimulant seed treatment, in a growth chamber experiment, by polymerase chain reaction-denaturing gradient gel electrophoresis of partial 16S rRNA gene and amplicon sequencing. Cluster analyses showed that the biostimulant treatment affected the rhizosphere bacterial microbiota of the ordinary hybrid more than that of the early vigor, both at plant emergence and at the 5-leaf stage. Moreover, the diversity indices calculated from the community profiles, revealed significant effects of NP fertilization on richness and the estimated effective number of species (*H_2_*) in both maize genotypes, while the biostimulant had a positive effect on plant growth promoting community of the ordinary hybrid, both at the plant emergence and at the fifth leaf stage. Our data showed that maize genotype was the major factor shaping rhizosphere bacterial community composition suggesting that the root system of the two maize hybrids recruited a different microbiota. Moreover, for the first time, we identified at the species and genus level the predominant native bacteria associated with two maize hybrids differing for vigor. These results pave the way for further studies to be performed on the effects of cropping system and specific crop practices, considering also the application of biostimulants, on beneficial rhizosphere microorganisms.

## 1 Introduction

Maize (*Zea mays* L.) is one of the most important crops worldwide, with an annual average production of 1115 million tonnes (FAOSTAT, 2022), destined to several sectors, with a particular rising use in gluten free food and industrial (starch industry) or energy purpose (García-Lara and Serna-Saldivar, 2019). The success of maize is related to the high productive efficiency in the use of agronomic inputs, with a marked response to the applied agronomic practices. Within the crop practices, the sowing time, and particular an early planting date, play a key role, in temperate growing areas, in achieving the highest profitability of maize, due to an increase in the length of the growing cycle and in the solar radiation interception (Long et al., 2017) and then the possibility to sown long maturity hybrids. In addition, an early planting could lead to a lower risk of environmental stresses, such as drought and heat (Waqas et al., 2021) and higher grain quality and safety for a reduced mycotoxin level (Blandino et al., 2017). Furthermore, an early sowing date increased the risk to meet cold and rainy period during the maize emergence and the first vegetative stages, resulting in a slow plant development, with higher risk of damping-off, insect damages and weed competition, thus reducing the beneficial effects of an early sowing. The cultivation of hybrids with a superior tolerance to low temperatures, therefore, characterized by a high early vigor, instead of ordinary one (Peter et al., 2009; Reis et al., 2022), and the application at sowing of starter fertilizers in bands close to seed furrows (Ma et al., 2015; Kaiser et al., 2016), are the main crop practices applied to limit the risk of maize slow development within the early planting times. In fact, although agricultural soils may contain large amounts of total nitrogen (N) and phosphorus (P), they are mainly in a form not available to the plant (Imran et al., 2013), while cool springs could further reduce plant uptake of these nutrients in the early vegetative stages, limiting crop growth rate and leaf chlorophyll content (Zhao et al., 2022). Blandino et al. (2022) reported a synergistic effect of N and P applied as diammonium phosphate (DAP) in sub-surface band at sowing in the increase of maize early vigor and grain yield, even in soils with high N and P concentrations. Furthermore, the excessive use of fertilizers was found to increase greenhouse-gas emissions (Robertson and Vitousek, 2009) and to have potential negative impacts on soil health and long-term soil fertility, causing soil acidification (Juo et al., 1995; Matsuyama et al., 2005; Guo et al., 2010), reducing diversity in native microbial communities (Lazcano et al., 2013; Sun et al., 2015) and accelerating the eutrophication of water bodies (Carpenter et al., 1998; Withers and Haygarth, 2007). Due to the potential environmental pollution and the low crop uptake at early growth stages, the need for additional N and P fertilizer applications to soils with a high availability in these nutrients is uncertain (Schröder et al., 2015). Moreover, the policy and consumer demand for a more sustainable food and feed production stimulate studies aimed at developing resilient environmental-friendly cropping systems with a reduced application of external inputs, such as non-renewable fertilizers. An expression of this request is the Farm to Fork program of the EU Commission, which will require a reduction of nutrient losses by 50% and a decrease of the use of synthetic fertilisers by 20% by 2030 (European Commission Communication COM/2020/381).

A sustainable tool for the management of cropping system could be the valorisation of soil microbial communities. A strikingly high taxonomic bacterial diversity is estimated to reach a density range of 10^8^-10^10^ colony forming unit (CFU) g^-1^ dry soil (Roesch et al., 2007; Zhang et al., 2017). Compared to bulk soil, distinct bacterial communities live associated with plant roots, in the rhizosphere, affecting plant functions and productivity (van der Heijden et al., 2008; Mendes et al., 2013; Philippot et al., 2013). Many of the rhizosphere bacteria may enter in the functional category of plant growth promoting bacteria (PGPB), possessing specific metabolic traits enabling them to improve plant nutrient status and resistance to numerous biotic and abiotic stresses: *via* N fixation, phosphate and potassium solubilization, production of phytohormones, volatile organic carbon compounds (VOCs), siderophores, protective enzymes, such as chitinase, aminocyclopropane-1-carboxylic acid (ACC)-deaminase, induction of systemic resistance (ISR) and release of various antimicrobial substances (van der Heijden et al., 2008; Berg, 2009; Hayat et al., 2010; Mendes et al., 2013; Gouda et al., 2018). Some studies have reported that the interaction between maize genotype and specific agronomic management could impact the microbiota composition, richness and functionality (Favela et al., 2021). The recruitment of soil bacteria in the rhizosphere and endosphere was affected by plant genotype in diverse crops, such as potato, bean, rice and durum wheat (Manter et al., 2010; Shenton et al., 2016; Pérez-Jaramillo et al., 2017; Agnolucci et al., 2019; Ujvári et al., 2021) which was ascribed to differences in root architecture and rhizodeposition patterns (Bais et al., 2006; Badri and Vivanco, 2009). Large differences in rhizosphere microbial community composition were found among 27 maize hybrids and lineages (Peiffer et al., 2013; Walters et al., 2018), while qualitative differences in root colonization by bacterial endophytes were detected in different genotypes (Ikeda et al., 2013). However, the role played by hybrids with different agronomic attitude under stress conditions, such as the early vigor trait, in the regulation of plant-microorganism interactions has been less studied. P and N fertilization also proved to affect the composition and diversity of microbial communities occurring in maize rhizosphere and root endosphere (Zhu et al., 2016; Gomes et al., 2018; Miranda-Carrazco et al., 2022). In particular, root exudates, which are influenced by plant species, genotype and fertilization regime, are able to affect the rhizosphere microbial community composition and functionality (Sasse et al., 2018). Recently, the application of substances highly available and rapidly assimilated by soil microorganisms has been proposed as a way to enhance rhizosphere microbial community activity.

Moreover, several PGPB are today proposed to be applied as biostimulants and biofertilizers to the soil surface, in the seed furrow or to the seeds, in order to improve nutrient use efficiency or availability, while reducing agrochemical inputs, within a more sustainable crop management (Ruzzi and Aroca, 2015; Zaidi et al., 2015; Rouphael and Colla, 2020). Many bacterial taxa have been isolated and successfully used as PGPB and biostimulants, such as strains of the species *Azospirillum* sp. (Hungria et al., 2010), *Pantoea* sp. (Mishra et al., 2011), *Rhizobium* sp. (Chabot et al., 1996), *Serratia* sp. (Hameeda et al., 2006), *Pseudomonas* sp. (Kavino et al., 2010), *Paraburkholderia* sp. (Rahman et al., 2018), *Bacillus* sp. (Amaresan et al., 2019), *Lactobacillus* sp. (Shrestha et al., 2014), *Variovorax* sp. (Chandra et al., 2019) and *Ensifer meliloti* (Velásquez et al., 2020). PGP *Bacillus* species are considered optimal targets for bioinoculant development due to their distinctive trait of endospore formation, which results in longer product shelf-life, comparable with that of conventional agrochemicals (Qiao et al., 2014). *Bacillus amyloliquefaciens*, in particular, showed remarkable potentials for agricultural use (Qiao et al., 2014; Luo et al., 2022). Plant-associated strains of *B. amyloliquefaciens* demonstrated P solubilizing and N mineralizing abilities (Idriss et al., 2002; Hui et al., 2018), indole-3-acetic acid (IAA), cytokinin and ACC-deaminase production, siderophores, VOCs and several antifungal, antiviral and antibacterial secondary metabolites synthesis (Idris et al., 2007; Chen et al., 2009; Wang et al., 2016; Asari et al., 2017; Wu et al., 2019), as well as ameliorating capabilities through complex pathways in various stress conditions (Tiwari et al., 2017).

The effects of biostimulants inoculation on the complex habitat of the rhizosphere have not been adequately investigated in crop plants, also considering the interaction with other agronomic practices. The aim of this study was to monitor and characterize the native rhizosphere microbiota during the early growth phases of two maize genotypes, using a NP starter fertilization treatment and a biostimulant seed treatment, in a growth chamber experiment. To this aim, we assessed the diversity and composition of rhizosphere bacterial communities utilizing a culture-independent approach, such as PCR-DGGE (polymerase chain reaction – denaturing gradient gel electrophoresis) analysis of the 16S ribosomal RNA (rRNA) gene and amplicon sequencing.

A companion manuscript (see Part II) will report the effect of biostimulant seed treatment, NP starter fertilizer, genotype early vigor and their factorial combination, on maize development in the early stages and the consequential effect on growth, grain yield and quality, in growth chamber and open field experiments.

## 2 Materials and methods

### 2.1 Microcosm experiment

A growth chamber experiment was set up in order to investigate the effect of a seed biostimulant, based on a PGPB strain and a plant extract, on the diversity and composition of the bacterial communities of maize rhizosphere, also considering the interaction with genotypes with different early vigor and the application of NP starter fertilization in seed furrow.

Sixteen kilograms of natural silt loam sub-alkaline soil (Typic Ustifluvents, USDA classification) (Soil Survey Staff, 2010) were weighed and placed, after mixing it thoroughly, in each plastic pot (27 cm length × 24 cm width × 28 cm height). The soil was collected from the surface layer (0.2 m) in the field of the University of Turin experimental station, located in North-West Italy at Carmagnola (44° 53’ N, 7° 41’ E; elevation 245 m). The soil was characterized by a medium cation-exchange capacity (C.E.C.), low organic matter, potassium (K) and P content and medium nitrogen N availability. More information on soil physical and chemical parameters are reported in **Table S1**. Soil was not air dried, sieved, sterilized and mixed with quartz sand or other materials.

All maize seeds, independent from biostimulants treatment, were treated with a fungicide mixture of prothioconazole (100 g L^-1^) and metalaxyl (20 g L^-1^) applied at 15 mL to 50,000 seeds (Redigo^®^ M, Bayer Crop Science S.r.l., Monheim am Rhein, Germany). Maize seeds shape, dimension and weight were carefully chosen in order to reduce seedling vigor variability. In each pot, 4 maize seeds were sown by hand at 2 cm of depth, equally distributed. NP fertilizer was placed manually in a hypothetical seed furrow band, 5 cm close to maize seed furrows, at a depth of 10 cm. No other fertilizers were applied before or after sowing.

Pots were placed in a controlled growth chamber with 50% relative humidity range, 12 h photoperiod, 700 μmol m^-2^ s^-1^ photosynthetically active radiation (PAR) and 14/17°C (night/day) air temperature range (**Table S2**). The air and soil temperatures have been controlled during the experiment by means of two data loggers: HOBO^®^ Pro v2 (Onset Computer Corp., Bourne, MA, USA) and Tinytag Plus 2 GP-4020 with 10 cm thermistor probe (Gemini Data Loggers Ltd, Chichester, UK), respectively.

Soil moisture content was maintained at water holding capacity by adding weekly in each pot 0.57 L of water, corresponding to 10 mm of rain. The weed control was carried out manually to eliminate every undesired plant seedling just after germination. The experiment was terminated 49 days after sowing (DAS).

### 2.2 Experimental design

The compared treatments were factorial combinations of:

 • maize hybrids, considering genotypes with different early vigor after emergence but with similar growing cycle (FAO maturity class 600, 130 maturity days),
  ◦ an ordinary hybrid (ordinary), with conventional early vigor (LG30600, Limagrain Europe, Saint-Beauzire, France),
  ◦ a high early vigor hybrid (high early vigor), with a rapid growth in the first vegetative stages (LG31630, characterized by the Rapid’START trait, Limagrain Europe);
 • NP starter fertilization,
  ◦ unfertilized control (unfertilized),
  ◦ sub-surface starter fertilization (NP), 27 kg N ha^-1^ and 69 kg P_2_O_5_ ha^-1^ were applied as diammonium phosphate (DAP, 18% and 46% for N and P_2_O_5_, respectively w/w) placed in bands close to the maize seed furrow;
 • biostimulant seed treatment,
  ◦ untreated control (no biostimulant),
  ◦ biostimulant seed application (biostimulant, commercial product Starcover, Limagrain Europe), based on a mixture of a bacterium, *Bacillus amyloliquefaciens* strain IT-45 (Rise P^®^ Lallemand Plant Care, Castelmaurou, France) and a leguminous plant extract *Cyamopsis psoraloides* (AgRHO^®^ GSB30 Solvay, Clamecy, France) which works as coating film to favor germination by channeling water from soil to seed.

The experimental design was a completely randomized block design with three replications.

### 2.3 Sample collection and preparation

Maize plants were harvested at 13 (emergence) and 49 (5-leaf stage) DAS. Since at emergence the seedling is not still reaching the band where the NP starter fertilizer is placed, at this growth stage, plants were harvested only from unfertilized pot. At 5-leaf stage all the compared factors were considered. At each growth stage, the whole roots system of 2 plants was collected after cutting maize shoots at the collar and gently removing the soil by hand. Each treatment was represented by triplicate rhizosphere samples collected from separate pot cultures (2 plants per pot). Samples were stored on 4°C until further analysis. Rhizosphere samples were separated from the roots in sterile Falcon tubes, adding 40 mL sterile physiologic solution (0.9% (w/v) NaCl; 0.005% (w/v) Tween80) to each sample and shaking them in a Lab-Line^®^ Multi-Wrist™ shaker (Lab-Line Instruments, Melrose Park, IL, USA). After 10 minutes of shaking, clean roots were extracted from the solution. The remaining soil was centrifuged on 5500 rpm for 10 minutes, and the supernatant was eliminated.

### 2.4 DNA extraction

250 mg subsamples of rhizosphere soil were subjected to genomic DNA extraction using the DNEasy^®^ Power Soil^®^ Kit (Qiagen GmbH, Hilden, Germany), following the manufacturer’s instructions. The extracted DNA was stored at −20°C and subsequently used for the molecular analysis of soil bacterial communities.

### 2.5 Molecular analysis of bacterial community profiles with PCR-DGGE

For PCR-DGGE, the V3-V5 hypervariable region of the 16S rDNA was amplified. PCR was carried out using the primers 341F (5’-CCT ACG GGA GGC AGC AG-3’) and 907R (5’-CCG TCA ATT CCT TTR AG TTT-3’) (Eurofins, Ebersberg, Germany) (Yu and Morrison, 2004). The primer 341F had an additional 40-nucleotide GC-rich tail (5’-CGC CCG CCG CGC CCC GCG CCC GTC CCG CCG CCC CCG CCC G-3’) on the 5’ end to prevent complete DNA denaturation during the DGGE process.

Reaction mixes were prepared in a final volume of 50 μL, containing 1 μL of 1:100 diluted DNA extract (10–20 ng of DNA). Each reaction mixture contained 5 μL of ExTaq Buffer 10x (Takara Bio Inc., Kusatsu, Japan), 1.25 U of ExTaq (Takara Bio Inc.), 0.2 mM of each dNTP (Takara Bio Inc.) and 0.5 μM of both primers. The reaction was carried out in an iCycler-iQ™ Multicolor Real-Time PCR Detection System (Bio-Rad, Hercules, CA, USA) with the following thermal cycles: initial denaturation at 94°C for 1’; 35 cycles of denaturation – annealing – elongation at 94°C for 30”, at 60°C for 30” and at 72°C for 30”, respectively; and final elongation at 72°C for 5’.

The expected product was about 560 bp long. The presence of amplicons was confirmed by electrophoresis in 1.5% (w/v) agarose gels in 1x TBE buffer (Tris-borate-EDTA, pH 8.3) (AppliChem GmbH, Darmstadt, Germany) stained with 20000x RealSafe Nucleic Acid Staining Solution (Durviz s.l., Valencia, Spain). DNA fragments were visualized over an UV transilluminator (Uvitec Cambridge, Cambridge, UK), and pictures were captured with the UVI 1D v. 16.11 program (Uvitec Cambridge) in TIFF format.

For molecular analysis of the bacterial diversity, 20 μL of amplicon DNA was separated in 8% (w/v) polyacrylamide 4K (AppliChem GmbH) gels in the DCode™ Universal Mutation Detection System (Bio-Rad). The urea-formamide denaturing gradient was 36-52%. An unfertilized/no biostimulant sample of the ordinary hybrid at the 5-leaf stage was loaded on both sides and in the middle of the gels as marker. Gels were run at 80 V for 16 h in 1x TAE buffer (Tris-acetate-EDTA, pH 8.5) (AppliChem GmbH) at 60°C. Subsequently, gels were stained in 1x TAE buffer with 10000x SYBR Gold Nucleic Acid Gel Stain (Thermo Fischer Scientific, Waltham, MA, USA) and visualized over an UV transilluminator as described above.

### 2.6 DGGE profile analysis

DGGE profiles were digitally processed with the BioNumerics software v. 8.1 (Applied Maths, St-Martens-Latem, Belgium) as reported in Turrini et al. (2017). Sample profiles were normalised to contain the same extent of total signal after background subtraction, and lanes were straightened and aligned following the manufacturer’s instructions. Markers were used for further normalisation between separate gels allowing their comparison. Bands were designated by manual supervision of the auto search bands function, and band positions were converted to Rf% values. Similarities between DGGE profiles were calculated with Pearson’s similarity coefficients applied on the lane patterns using the band-matching tool with 0% of optimization. The similarity coefficients were then used for generating dendrograms with the Unweighted Pair-Group Method Using Arithmetic Average (UPGMA) cluster analysis tool.

Based on the banding data and treating each band as an individual operational taxonomic unit (OTU), six different diversity indices were calculated. Richness (*S*) indicated the number of OTUs detected in the sample. Shannon-Weaver’s diversity (*H_s_*) and Simpson’s dominance (*D*) indices were calculated as $\;H_{s}=\sum_{i=1}^{n}-\frac{h_{i}}{H}\cdot \ln\frac{h_{i}}{H}$ and $\;D=\sum_{i=1}^{n}\frac{h_{i}\cdot (h_{i}-1)}{H\cdot (H-1)}$ , where *h_i_* was the peak intensity of a band and *H* was the sum of all peak intensities in a sample. Evenness (*J_p_*) allowed to reveal the presence of dominant OTUs, calculated as $J_{p}=\frac{H_{s}}{\ln S}$ . Hill 1 (*H_1_*) and Hill 2 (*H_2_*) numbers were computed as $H_{1}=\frac{1}{D}$ and $H_{2}=e^{H_{s}}$ , respectively.

### 2.7 DGGE band sequencing

The main bands of the DGGE profiles were cut from the gel for further molecular analysis. Bands were eluted in 50 μL UltraPure™ DNase/RNase-free distilled water (Invitrogen, Waltham, MA, USA) for three days at 4°C. Supernatants were diluted 1:100 and served as templates for PCR using the primers 341F and 907R without GC-clamp, following the protocol described earlier. PCR products were then purified with the QIAquick^®^ PCR Purification kit (Qiagen GmbH) according to the manufacturer’s instructions. Purified amplicons were eluted in 50 μL H_2_O and controlled in a 2% agarose gel to confirm product quality, and their concentration for dsDNA was estimated with an Eppendorf Biophotometer (Eppendorf SE, Hamburg, Germany) measuring at λ= 260 nm. Partial 16S rDNA amplicons were 5’-end sequenced by Eurofins Genomics – Mix2Seq Custom DNA Sequencing Services (Ebersberg, Germany). Sequences were analysed as in Palla et al. (2022), using BLAST (https://blast.ncbi.nlm.nih.gov/Blast.cgi) in the NCBI-GenBank (https://www.ncbi.nlm.nih.gov/genbank) database, accessed in July, 2022. Related sequences were collected and aligned with the MUSCLE tool (Edgar, 2004a; Edgar, 2004b) in the MEGA 11 software (Tamura et al., 2021). Phylogenetic trees were constructed using the Maximum Likelihood method based on Kimura’s 2-parameter model (Kimura, 1980) in MEGA 11 with 1000 bootstrap replicates. The DGGE band sequences were submitted to the NCBI-GenBank database under the accession numbers from OP964519 to OP964570; OP985320; OQ000256.

### 2.8 Statistical analysis

Statistical analyses were carried out with the SPSS v. 25 software (IBM Corp., Armonk, NY, USA). Homogeneity of variances was controlled with Levene’s test (p< 0.05) and data transformed, if needed. Two-ways analysis of variances (ANOVA) was conducted on the diversity indices obtained from DGGE profiles of rhizosphere samples at the emergence stage considering the hybrid and biostimulant treatment, which have an influence on plant at this growth stage, as factors, each at two levels. Three-ways ANOVA was carried out on the diversity indices of 5-leaf stage rhizosphere samples considering the hybrid, NP starter fertilization and biostimulant seed treatment as factors, each at two levels. Hill1 and Evenness indices showed heterogeneous variances even after transformation and were analysed by two-way ANOVA, considering NP starter fertilization and biostimulant seed treatment as factors for the two hybrids separately.

## 3 Results

### 3.1 Analysis of PCR-DGGE profiles

**Figure 1:**
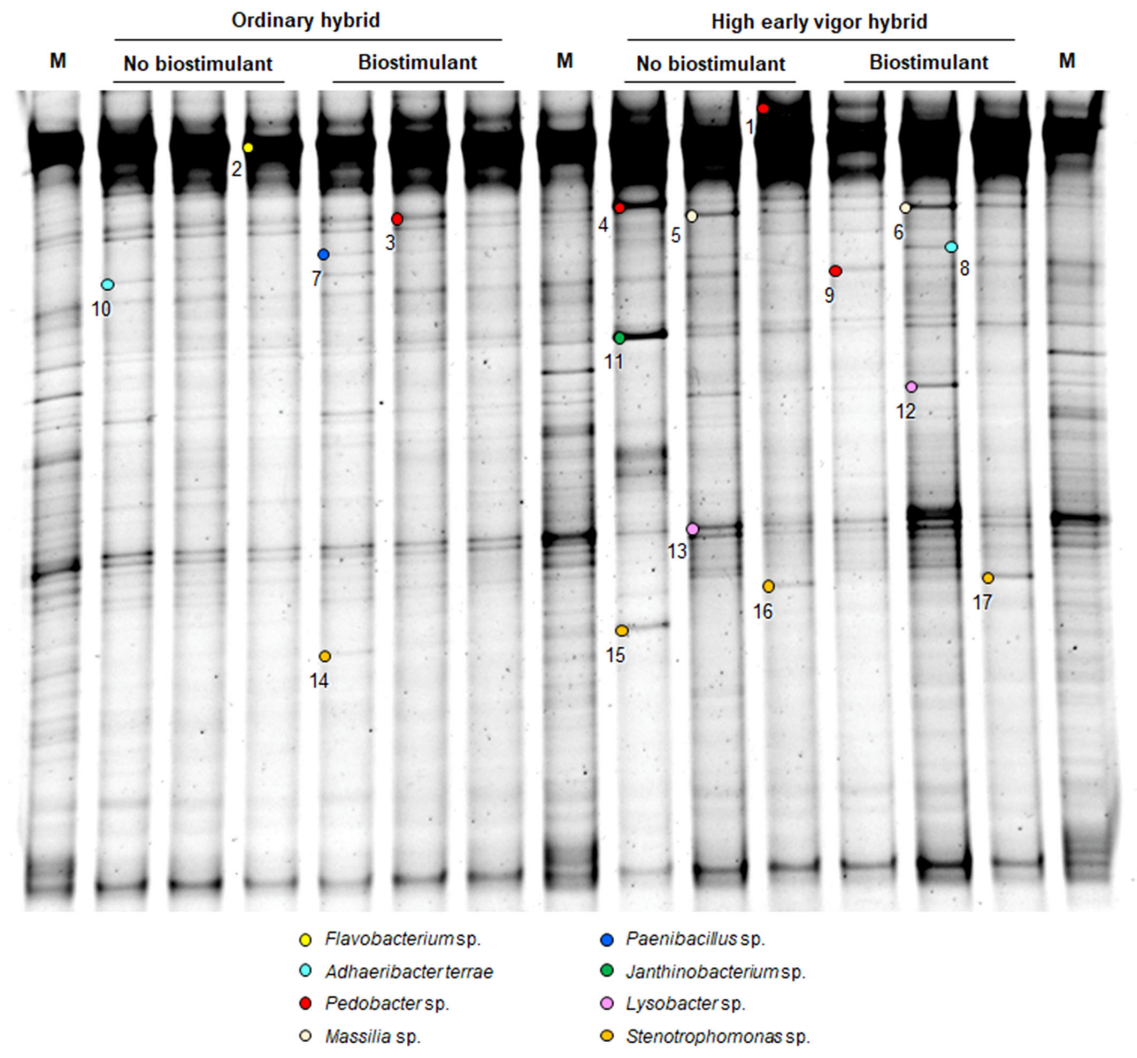
PCR-DGGE profiles of the rhizosphere bacterial communities of two maize hybrids at the emergence stage, treated or untreated with seed-applied biostimulant. Marker: M. The numbers indicate sequenced DNA fragments and the colored circles the relevant bacterial species affiliation.

**Figure 2:**
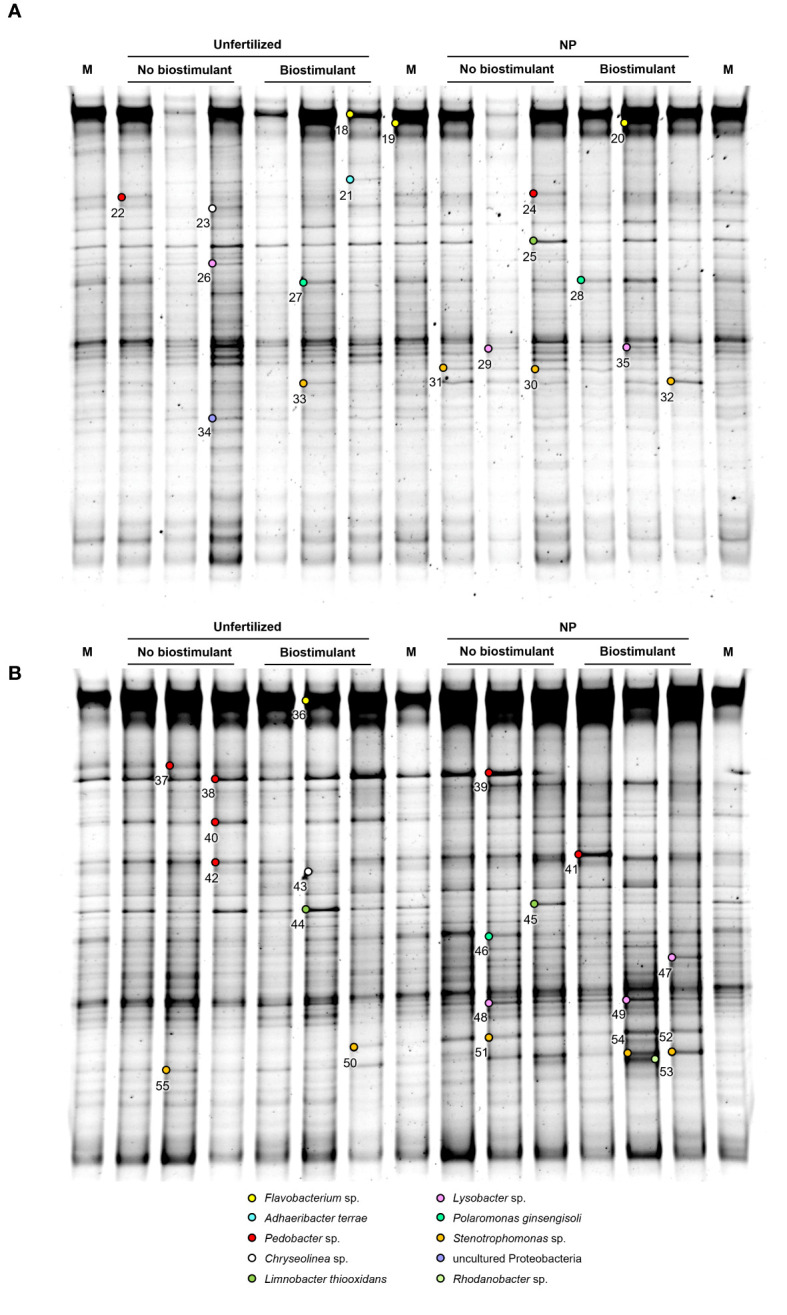
PCR-DGGE profiles of the rhizosphere bacterial communities of two maize hybrids at the 5-leaf stage, treated or untreated with NP starter fertilization and with seed applied biostimulant. **(A)** ordinary hybrid. **(B)** high early vigor hybrid. M: Marker. The numbers indicate sequenced DNA fragments and the colored circles the relevant bacterial species affiliation.

Bacterial 16S rDNA fragments (ca. 560 bp) were successfully amplified in all samples. The DGGE separation of the PCR amplicons revealed rhizosphere bacterial community profiles, characterized by a high number of bands of variable intensities (**Figures 1**, **2**). DGGE profiles were compared by cluster analysis (UPGMA), and biodiversity indices (*S*, *H_s_*, *D*, *J_p_*, *H_1_*, *H_2_*) were estimated based on the banding patterns.

**Figure 3:**
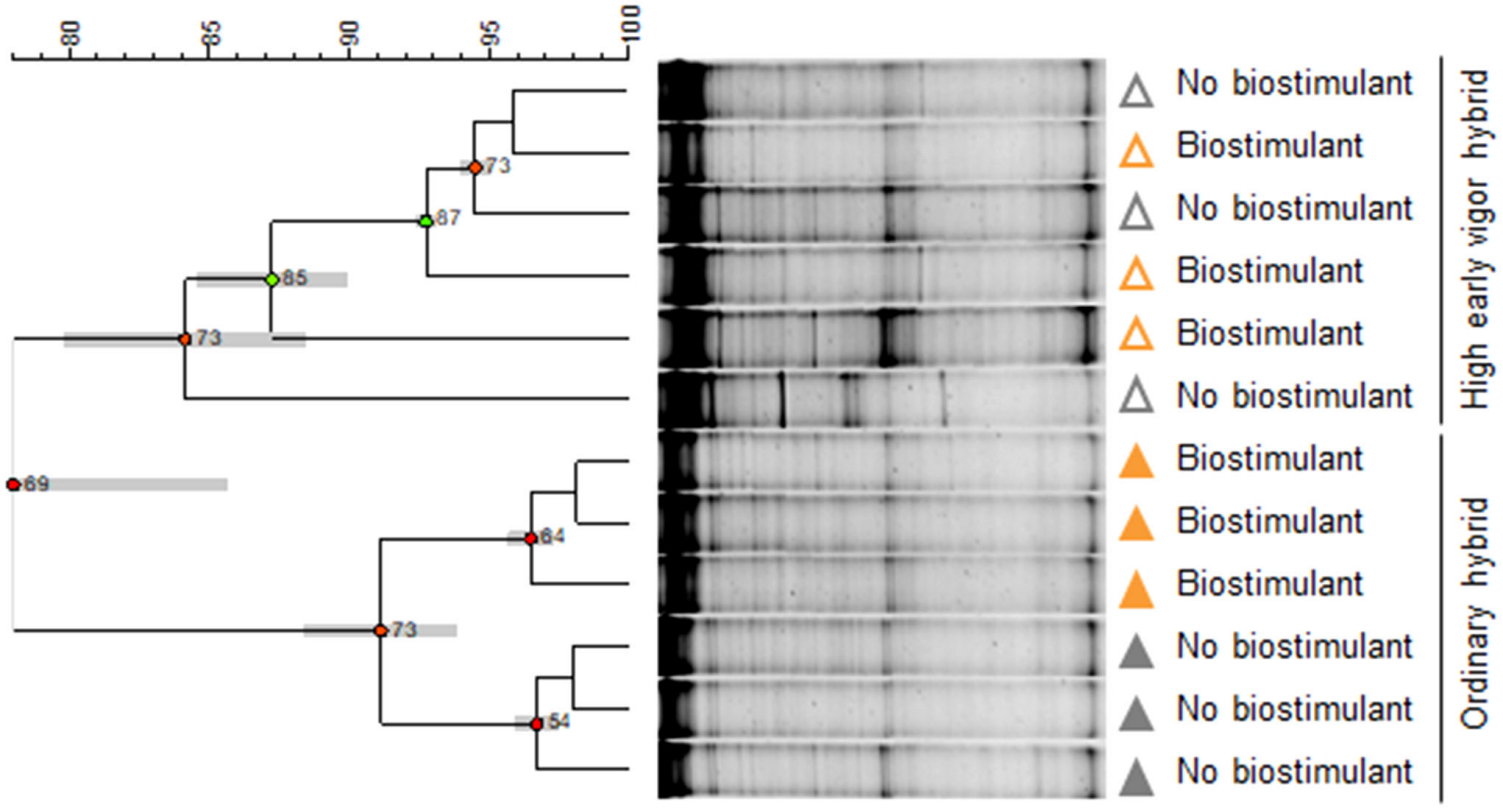
UPGMA (Unweighted Pair Group Method Using Arithmetic Average) cluster analysis of rhizosphere bacterial community DGGE profiles. Relationships among samples are based on Dice’s similarity coefficient, as shown by the numeric scale above each dendrogram. Dendrograms are based on DGGE profiles obtained from the rhizosphere of two maize hybrids at the emergence stage treated or untreated with seed applied biostimulant. Cophenetic correlation, expressing the consistency of clusters, is shown at each node by numbers and colored dots, ranging between green-yellow-orange-red, according to decreasing values. Standard deviation is shown at each node by a grey bar. Colors indicate the factorial treatments: unfertilized/no biostimulant (grey), biostimulant seed treatment (orange). Closed and open symbols refer to the ordinary and to the high early vigor maize hybrids, respectively.

At the emergence stage, the rhizosphere bacterial communities of the two maize hybrids clustered separately in the UPGMA dendrogram (**Figure 3**), with a similarity of 78%. Interestingly, in the ordinary hybrid, samples treated with seed-applied biostimulant clustered separately from the control (91% similarity), while there was no such a separation in the high early vigor hybrid.

**Figure 4:**
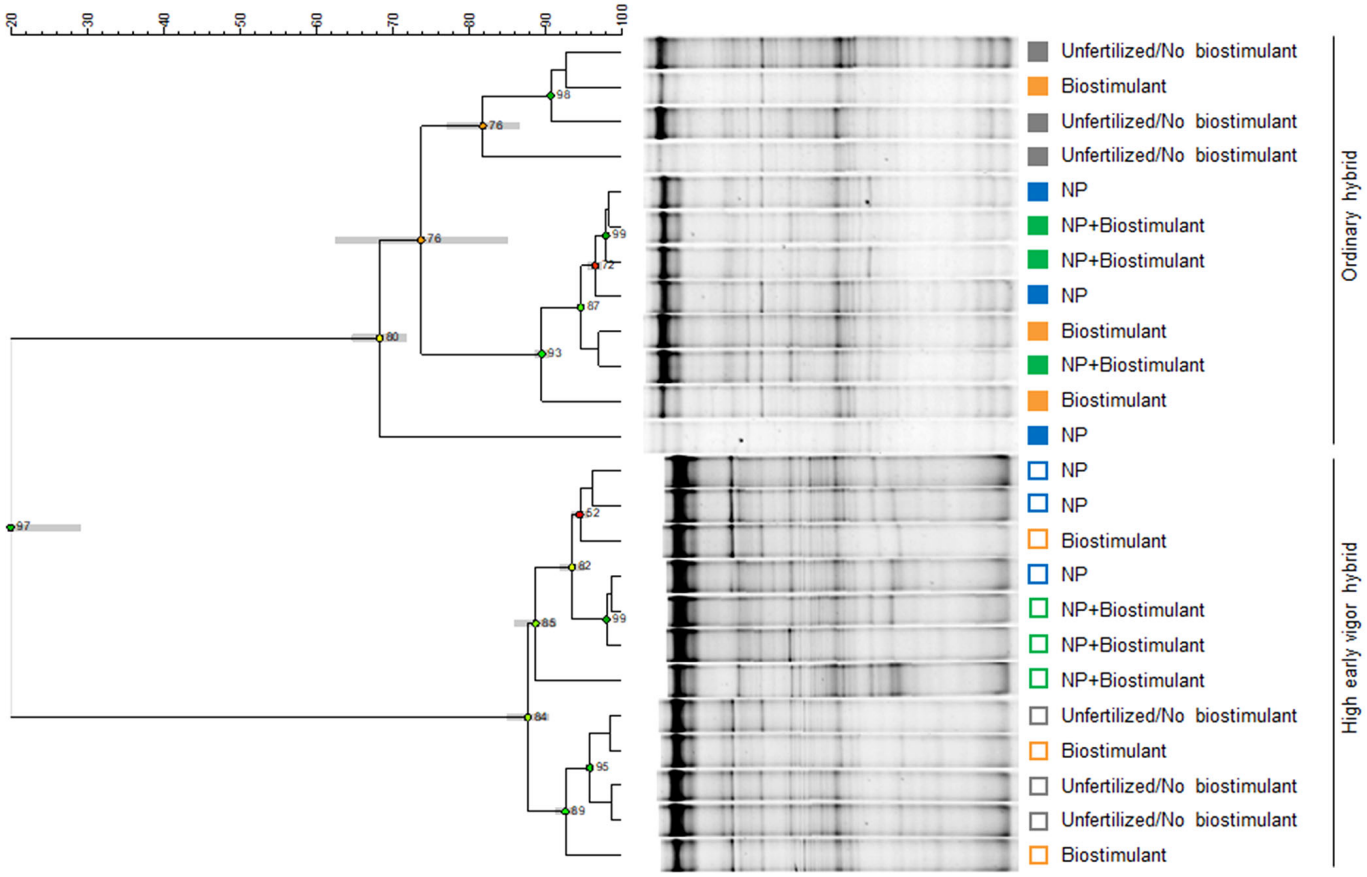
UPGMA (Unweighted Pair Group Method Using Arithmetic Average) cluster analysis of rhizosphere bacterial community DGGE profiles. Relationships among samples are based on Dice’s similarity coefficient, as shown by the numeric scale above each dendrogram. Dendrograms are based on DGGE profiles obtained from the rhizosphere of two maize hybrids at the 5-leaf stage treated or untreated with NP starter fertilization and with seed-applied biostimulant. Cophenetic correlation, expressing the consistency of clusters, is shown at each node by numbers and colored dots, ranging between green-yellow-orange-red, according to decreasing values. Standard deviation is shown at each node by a grey bar. Colors indicate the factorial treatments: unfertilized/no biostimulant (grey), biostimulant seed treatment (orange), NP starter fertilization (blue) and NP + biostimulant (green). Closed and open symbols refer to the ordinary and to the high early vigor maize hybrids, respectively.

Two-ways ANOVA of the diversity indices revealed the early effect of the genotype on richness, which was higher in the ordinary hybrid, compared with the high early vigor hybrid, while biostimulant treatment did not significantly affect bacterial diversity at the time of emergence (**Table 1**). As a result of the UPGMA cluster analysis of the 5-leaf stage samples (**Figure 4**), the two hybrids grouped separately with a very low similarity value (20%). Within both maize genotypes, unfertilized/no biostimulant samples clustered separately from those treated with NP fertilizer, showing similarities lower than 74% and 88% for the ordinary hybrid and the high early vigor hybrid, respectively.

**Table 1:** Effects of the hybrid and biostimulant seed treatment on diversity indices calculated from bacterial 16S rDNA DGGE profiles of the rhizosphere samples at the emergence stage.

Factor	Source of variation	Richness (*S*) ± SD	Hill 2 (*H_2_*) ± SD	Hill 1 (*H_1_*) ± SD	Evenness (*J_p_*) ± SD
Hybrid (H)	Ordinary	12.67 ± 1.21 a	9.67 ± 1.25	8.09 ± 1.32	0.89 ± 0.02
	High early vigor	10.83 ± 1.47 b	8.37 ± 1.15	7.03 ± 1.21	0.89 ± 0.03
	*p*-value	0.039	0.106	0.198	0.937
Seed treatment (S)	No biostimulant	11.50 ± 1.87	8.93 ± 1.72	7.54 ± 1.80	0.89 ± 0.03
	Biostimulant	12.00 ± 1.41	9.10 ± 0.95	7.59 ± 0.78	0.89 ± 0.02
	*p*-value	0.521	0.811	0.943	0.804
H x S	*p*-value	0.156	0.275	0.289	0.960

Means followed by different letters are significantly different. The level of significance (*p*-value) is shown in the Table. The data reported for each factor are based on 6 observations ± standard deviation (SD).

Concurrently, analysing the diversity indices calculated from the community profiles, three-ways ANOVA revealed significant effects of hybrid and NP fertilization on richness and *H_2_*, with higher diversity values in the high early vigor hybrid and NP fertilized samples (**Table 2**). As variances were not homogeneous, *H_1_* and evenness indices were analysed by two-ways ANOVA, which showed significant increases induced by fertilization in the ordinary hybrid, but not in the high early vigor hybrid (**Table 3**). By contrast, seed-applied biostimulant did not influence any of the biodiversity indices.

**Table 2:** Effects of the hybrid, NP starter fertilization and biostimulant seed treatment on diversity indices calculated from bacterial 16S rDNA DGGE profiles of the rhizosphere samples at the 5-leaf stage.

Factor	Source of variation	Richness (*S*) ± SD	Hill 2 (*H_2_*) ± SD
Hybrid (H)	Ordinary	11.83 ± 1.64 b	9.95 ± 1.53 b
	High early vigor	17.00 ± 1.91 a	13.32 ± 2.30 a
	*p*-value	< 0.001	< 0.001
Fertilization (F)	Unfertilized	13.08 ± 2.71 b	10.34 ± 1.99 b
	NP	15.75 ± 3.11 a	12.93 ± 2.50 a
	*p*-value	< 0.001	0.001
Seed treatment (S)	No biostimulant	14.25 ± 3.20	11.57 ± 2.53
	Biostimulant	14.58 ± 3.26	11.70 ± 2.73
	*p*-value	0.490	0.928
H x S	*p*-value	1.000	0.834
H x F	*p*-value	0.490	0.908
F x S	*p*-value	0.728	0.231
H x F x S	*p*-value	0.096	0.772

Means followed by different letters are significantly different. The level of significance (*p*-value) is shown in the Table. The data reported for each factor are based on 12 observations ± standard deviation (SD).

**Table 3:** Effects of NP starter fertilization and biostimulant seed treatment on diversity indices calculated from bacterial 16S rDNA DGGE profiles of the rhizosphere samples at the 5-leaf stage for each hybrid.

**Factor**	**Source of variation**	**Hill 1 (*H_1_*) ± SD**		**Evenness (*J_p_*) ± SD**	
		**Ordinary hybrid**	**High early vigor hybrid**	**Ordinary hybrid**	**High early vigor hybrid**
Fertilization (F)	Unfertilized	7.82 ± 0.85 b	9.78 ± 2.03	0.92 ± 0.02 b	0.90 ± 0.04
	NP	10.12 ± 0.72 a	12.53 ± 3.16	0.94 ± 0.01 a	0.92 ± 0.04
	*p*-value	0.001	0.131	0.044	0.379
Seed treatment (S)	No biostimulant	8.80 ± 1.03	11.17 ± 2.86	0.93 ± 0.01	0.92 ± 0.04
	Biostimulant	9.14 ± 1.81	11.14 ± 3.24	0.93 ± 0.02	0.91 ± 0.04
	*p*-value	0.471	0.987	0.846	0.648
F x S	*p-*value	0.245	0.404	0.869	0.188

Means followed by different letters are significantly different. The level of significance (*p*-value) is shown in the Table. The data reported for each factor are based on 6 observations ± standard deviation (SD).

### 3.2 DGGE amplicon sequencing and identification of the main bacterial taxa

**Figure 5:**
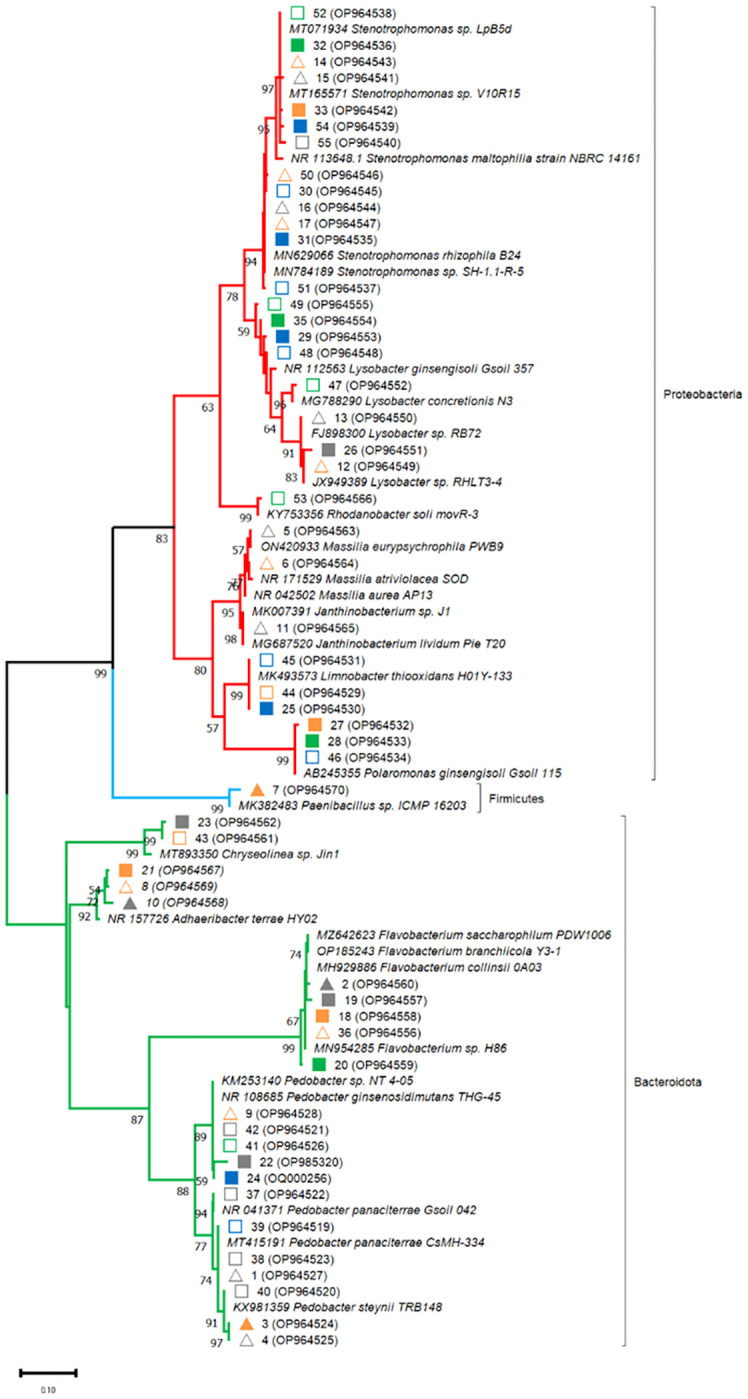
Affiliation of the sequences retrieved from DGGE gel fragments (marked in **Figures 2** and **4**) with the sequences of the 16S rRNA gene retrieved in gene banks. Phylogenetic analysis was inferred by using the Maximum Likelihood method. The evolutionary distances were computed using the Kimura 2-parameter model. Bootstrap (1000 replicates) values below 70 are not shown. Evolutionary analyses were conducted in MEGA11.The sequences from the database are indicated by their accession numbers. The DNA sequences retrieved in this work are indicated by their corresponding band number and their accession number. Symbols indicate samples analysed at the emergence stage (triangles) and at the 5-leaf stage (squares). Closed and open symbols refer to the ordinary and to the high early vigor maize hybrids, respectively. Colors indicate the factorial treatments: unfertilized/no biostimulant (grey), biostimulant (orange), NP starter fertilization (blue) and NP + biostimulant (green).

In order to identify major bacterial taxa characterizing the rhizosphere soils of different maize hybrids in the factorial combinations of fertilizer and biostimulant treatments, relevant bands were excised from DGGE gels (**Figures 1** and **2**), sequenced and affiliated to genera and species by using nBLAST and phylogenetic tree analyses. Partial 16S rDNA fragments belonged to three phyla, namely Proteobacteria (*Stenotrophomonas* sp., *Lysobacter* sp., *Polaromonas ginsengisoli*, *Limnobacter thiooxidans*, *Massilia* sp., *Rhodanobacter* sp., *Janthinobacterium* sp., uncultured Proteobacteria), Bacteroidetes (*Flavobacterium* sp., *Pedobacter* sp., *Chryseolinea* sp., *Adhaeribacter terrae*) and Firmicutes (*Paenibacillus* sp.) (**Table S3**; **Figure 5**). None of the sequenced bands affiliated with *B. amyloliquefaciens*.

16S rDNA fragments affiliating with *Massilia* sp. (5-6), *Paenibacillus* sp. (7) and *Janthinobacterium* sp. (11) could be recovered only from the emergence stage samples. At emergence stage, more variable and more intense banding patterns could be observed in the rhizosphere of the high early vigor hybrid, with occasional increases in the abundance of *Pedobacter* sp. (4), *Massilia* sp. (5-6), *Janthinobacterium* sp. (11), *Lysobacter* sp. (12-13) and *Stenotrophomonas* sp. (14-17), while bacterial communities showed much more uniform molecular profiles in the samples of the ordinary hybrid (**Figure 1**). Additionally, the higher abundance of the OTUs corresponding to *Paenibacillus* sp. (7) and *Stenotrophomonas* sp. (14) were associated with the biostimulant treatment in the ordinary hybrid.

Fragments corresponding to *Chryseolinea* sp. (23, 43), *Limnobacter thiooxidans* (25, 44-45), *Polaromonas ginsengisoli* (27-28, 46) and *Rhodanobacter* sp. (53) could be retrieved only from the 5-leaf stage rhizosphere samples. At the 5-leaf stage, despite most bacterial populations were represented uniformly in all samples of each hybrid, marginal fluctuations could be observed in the abundance of some taxa. Biostimulant application slightly increased the abundance of *Polaromonas ginsengisoli* (27-28) in the ordinary maize genotype, while other changes were detected mainly due to the NP fertilization treatment (**Figure 2**). In the ordinary hybrid, slightly higher abundance of *Limnobacter thiooxidans* (25), *Lysobacter* sp. (26) and an uncultured Proteobacteria (34) were associated to the unfertilized samples, while bands of *Stenotrophomonas* sp. (30-33) were more characteristic of the NP fertilization treatments. In the high early vigor hybrid, somewhat similar changes could be observed: bands affiliated with *Limnobacter thiooxidans* (44-45) appeared more intensely in the unfertilized samples, while *Stenotrophomonas* sp. (50-52, 54-55) remained more associated to the NP fertilizer treatments. Additionally, *Pedobacter steynii* (40) was more represented in the unfertilized maize rhizosphere, and *Polaromonas ginsengisoli* (46) and some *Lysobacter* sp. (48-49) in the NP fertilization treatments. Sequences affiliated to *Rhodanobacter* sp. (53) could be retrieved only from the NP fertilized samples of the high early vigor hybrid.

## 4 Discussion

Our data showed that maize genotype was the major factor shaping rhizosphere bacterial community composition, as assessed by cluster analyses of DGGE patterns, suggesting that the root system of the two maize hybrids recruited a different microbiota. Here, for the first time we identified at the species and genus level the predominant native bacteria associated with two maize hybrids differing for vigor. The biostimulant treatment affected the rhizosphere bacterial microbiota of the ordinary hybrid more than that of the early vigor, both at plant emergence and at the 5-leaf stage. Moreover, the 5-leaf stage rhizosphere bacterial community composition was differentially affected by starter NP fertilization, compared with that of the unfertilized/no biostimulant in both hybrids.

### 4.1 DGGE cluster analysis

Cluster analysis of the DGGE profiles detected significantly different rhizosphere bacterial community profiles among the two maize hybrids, which became more evident at the 5-leaf stage.

Differences in the rhizosphere microbiota of the two hybrids may be attributed to the influences of plant genotype on the assemblages of plant associated microbial communities (Manter et al., 2010; Shenton et al., 2016; Pérez-Jaramillo et al., 2017; Agnolucci et al., 2019). Genetical differences between cultivated crop varieties have been shown to affect root architecture, rhizodeposition patterns and plant-microbe signaling pathways (Hu et al., 2018; Kerstens et al., 2021; Semchenko et al., 2021). Rhizodeposition of sugars, organic acids, amino acids and secondary metabolites plays a crucial role in the recruitment and regulation of root associated microbiota, as some serve as signals, and some are easily available nutrients to heterotrophic bacteria (Philippot et al., 2013; Canarini et al., 2019).

Cluster analysis showed that seed-applied biostimulant preparations had contrasting effects on the two maize hybrids. Biostimulant treatment affected the rhizosphere bacterial microbiota of the ordinary hybrid since the emergence stage, which grouped separately from no biostimulant samples. Our data are consistent with those obtained in juvenile maize inoculated with *B. amyloliquefaciens* FZB42, revealing shifts in the PCR-DGGE rhizosphere bacterial community profiles, compared with uninoculated samples (Cozzolino et al., 2021). Furthermore, inoculation with *B. amyloliquefaciens* L-S60, B1408 and FZB42 caused changes in the rhizosphere bacterial communities of cucumber seedlings and tomato plants (Qin et al., 2017; Eltlbany et al., 2019; Han et al., 2019), while a biofertilizer preparation containing *B. amyloliquefaciens* NJN-6 had similar effects in field-grown banana plants (Shen et al., 2015). By contrast, our biostimulant treatment had marginal effects on the rhizosphere bacterial community of the high early vigor maize hybrid at both growth stages, consistently with previous findings obtained in field-grown wheat inoculated with a consortium of PGP *Azospirillum* spp., *Azoarcus* spp. and *Azorhizobium* spp. (Dal Cortivo et al., 2020), and in lettuce and soybean treated with *B. amyloliquefaciens* (Correa et al., 2009; Chowdhury et al., 2013; Kröber et al., 2014). The different behavior of the two maize genotypes may be ascribed to differential interactions between native rhizosphere bacteria and the biostimulant used in this study, which could affect the multipartite relationships in the rhizosphere microbiota. Accordingly, previous studies demonstrated differences in the compatibility of some crop genotypes with various microbial inocula in wheat (Agnolucci et al., 2019; Akbari et al., 2020), tomato (Tucci et al., 2011), potato (Higdon et al., 2020) and sugarcane (de Oliveira et al., 2006).

The UPGMA cluster analysis highlighted also differential effects of NP fertilization on the rhizosphere bacterial community composition at the 5-leaf stage, as fertilized samples clustered separately from unfertilized/no biostimulant samples in both hybrids. Mineral fertilization was previously found to impact root-associated microbial communities in several crop plants (Tang et al., 2016; Chen et al., 2019; Semenov et al., 2020), and to change the bacterial community composition in maize rhizosphere, including the abundance of important bacterial functional genes and PGPB groups (Zhu et al., 2016; Silva et al., 2017; Gomes et al., 2018; Wang et al., 2018). Besides the direct effects of increased mineral nutrient availability in fertilized soils, rhizosphere microbial communities may be affected by alterations of root morphology and root exudates quality and quantity, as the result of improved plant growth and nutrient status (Lu et al., 1999; Zhu et al., 2016; Chen et al., 2019).

At the emergence stage, bacterial OTU richness was significantly higher in the ordinary hybrid, while at the 5-leaf stage the high early vigor hybrid hosted a larger and more diverse rhizosphere bacterial community. N and P amendments had a positive effect on bacterial diversity indices, as OTU richness and the estimated “effective number of species” (*H_2_*) increased in both maize genotypes, while evenness and another estimator of “effective number of species” (*H_1_*) only in the ordinary hybrid, confirming previous data on the changes of biodiversity of the rhizosphere microbiota by mineral fertilization (Wang et al., 2018; Semenov et al., 2020).

### 4.2 Sequencing of predominant DGGE bands

The sequencing of the main DGGE bands allowed the detection of 13 taxa, all belonging to genera and species of widespread occurrence in soils. The high representation of Proteobacteria among the sequenced bands is not surprising, as this phylum was previously reported to be predominant in maize rhizosphere (Peiffer et al., 2013; Li et al., 2014; Silva et al., 2017; Gomes et al., 2018; Wang et al., 2018). It is interesting to note that in the ordinary hybrid treated with the biostimulant, some bacteria belonging to taxa reported as PGP, were more represented. In particular, *Paenibacillus* sp. and *Stenotrophomonas* sp. were more abundant in the rhizosphere of emergent plantlets and *Polaromonas ginsengisoli* in the 5-leaf stage samples. In agreement with our data, Eltlbany et al. (2019) reported increases in the population size of *Paenibacillus* sp. as a result of bacterial biostimulant treatments in the rhizosphere of tomato plants. *Stenotrophomonas* spp. have been widely detected in plant-associated bacterial communities (Ryan et al., 2009; Hayward et al., 2010) and were reported to inhabit the maize rhizosphere (Medina-de la Rosa et al., 2016; Qaisrani et al., 2019; Ercole et al., 2021; Guo et al., 2022). Several isolates belonging to this genus were shown to promote plant growth *via* N_2_-fixation, to solubilize P and to produce ACC-deaminase, plant hormones and siderophores, acting as stress protective agents (Yu et al., 2011; Alavi et al., 2013; Ghavami et al., 2017; Singh and Jha, 2017; Youseif, 2018; Ercole et al., 2021). In the present study, this taxon reached higher abundance also in the NP fertilized rhizosphere of both hybrids, suggesting a possible responsiveness to P fertilization levels, as reported by Guo et al. (2022) in maize rhizosphere.

Certain taxa were more represented in the early vigor hybrid, particularly in NP treated samples, such as *Lysobacter* sp., which occurred in diverse habitats including soils (Hayward et al., 2010) and was previously reported to colonize or even dominate maize rhizosphere (García-Salamanca et al., 2013; Li et al., 2014; Maarastawi et al., 2018). Interestingly, strains of *Lysobacter* sp. have shown multiple PGP activities *in vitro*, such as P solubilization, siderophores and antibiotics production with promising biocontrol potentials (Hayward et al., 2010; Gómez Expósito et al., 2015; Puopolo et al., 2018; Sharma et al., 2021). *Rhodanobacter* sp. was detected only in the rhizosphere of the NP fertilized high early vigor hybrid, in agreement with previous findings showing that maize genotype and inorganic fertilizers may strongly affect its abundance in the rhizosphere soil (Wen et al., 2017; Semenov et al., 2020). However, although its occurrence was reported in the rhizosphere of maize plants by other authors (Chen et al., 2021; Shen et al., 2021), little is known of *Rhodanobacter* metabolic traits (van den Heuvel et al., 2010; Kostka et al., 2012; Damo et al., 2022).

In this work, certain bacterial taxa occurred only at the emergence stage, such as *Janthinobacterium* sp., *Massilia* sp. (family Oxalobacteriaceae), consistently with previous findings describing such genera as dominant in maize rhizosphere at early growth stages, with a sharp decline during the vegetative growth (Li et al., 2014). Interestingly, an isolate of *Janthinobacterium* sp. was found to express antagonism against a wide range of plant pathogens (Yin et al., 2021), while strains of *Massilia* sp. revealed important PGP characteristics, such as the production of phosphatases, siderophores and IAA and antagonism against pathogens (Hrynkiewicz et al., 2010; Turnbull et al., 2012; Raths et al., 2020; Li et al., 2021).

The phylum Bacteroidetes was represented mostly by *Flavobacterium* and *Pedobacter* species. The genus *Flavobacterium* (family Flavobacteriaceae) was uniformly distributed in the different treatments, regardless sampling time and maize genotype, consistently with previous data reporting high abundance of this genus in maize rhizosphere (Li et al., 2014; Correa-Galeote et al., 2016; Yang et al., 2017). Isolates of *Flavobacterium* sp. showed PGP traits, such as P-solubilization, ACC-deaminase and IAA production, while they provided significant plant growth promotion in maize, disease suppression in pepper, onion and cucumber and improved drought stress tolerance in wheat (Sang and Kim, 2012; Gontia-Mishra et al., 2016; Gontia-Mishra et al., 2017; Youseif, 2018; Nishioka et al., 2019). Also, the genus *Pedobacter* (family Sphingobacteriaceae) was found in all our samples, consistently with their described global occurrence (Steyn et al., 1998; Yoon et al., 2007; Gordon et al., 2009).

Fragments of *B. amyloliquefaciens* 16S rDNA was not retrieved from the DGGE gels, suggesting that the biostimulant strain was not a dominant member of the maize rhizosphere microbiota. Unfortunately, it is not possible to compare our data with previous ones obtained with the same molecular method, as similar works utilizing *B. amyloliquefaciens* BNM122 and FZB42 for soybean and maize inoculation, respectively, and PCR-DGGE, did not perform the identification of the main DGGE bands (Correa et al., 2009; Cozzolino et al., 2021). Moreover, other studies, utilizing different methods to monitor the persistence of *B. amyloliquefaciens* strains, such as serial dilutions and plate counting, were carried out in the absence of native bacterial communities (Correa et al., 2009; Ben Abdallah et al., 2018). Overall, the studies aimed at verifying the root persistence of *B. amyloliquefaciens* reported significant decreases over the course of time (Chowdhury et al., 2013; Kröber et al., 2014).

In conclusion, this work showed that rhizosphere bacterial community composition of maize was mainly affected by the genotype, as, for the first time, we identified at the species and genus level the predominant native bacteria associated with the root systems of the two maize hybrids, differing for their early vigor. The predominant native bacteria belonged to well-known PGPB taxa, such as *Stenotrophomonas* sp., *Lysobacter* sp., *Massilia* sp., *Paenibacillus* sp. and *Flavobacterium* sp., which were reported to be able to solubilize P and to produce IAA, siderophores and antibiotics, providing significant plant growth promotion and disease suppression. The starter NP fertilization strongly affected PGP rhizosphere bacterial community composition of both maize hybrids at the 5-leaf stage compared with that of the unfertilized treatments, while the biostimulant treatment had a positive effect on PGP community of the ordinary hybrid more than that of the early vigor maize both at the plant emergence and at the fifth leaf stage.

These results pave the way for further studies to be performed on the effects of cropping system and specific crop practices, considering also the application of biostimulants, on beneficial rhizosphere microorganisms.

## Data availability statement

The datasets presented in this study can be found in online repositories. The names of the repository/repositories and accession number(s) can be found below: https://www.ncbi.nlm.nih.gov/genbank/, OP964519 to OP964570 https://www.ncbi.nlm.nih.gov/genbank/, OP985320 https://www.ncbi.nlm.nih.gov/genbank/, OQ000256.

## Author contributions

This work was conceptualized and supervised by MA, MG and MB. Methodology and investigation was done by GU, LC, AG, IP, LC and CC. Data was processed by GU, CC, MA and AT. The original draft was written by GU, MA and MG. All authors contributed to the article and approved the submitted version.

## References

[B1] Agnolucci M. Palla M. Cristani C. Cavallo N. Giovannetti M. De Angelis M.. (2019). Beneficial plant microorganisms affect the endophytic bacterial communities of durum wheat roots as detected by different molecular approaches. Front. Microbiol. 10. doi: 10.3389/fmicb.2019.02500 PMC6834690 31736925

[B2] Akbari A. Gharanjik S. Koobaz P. Sadeghi A. (2020). Plant growth promoting *Streptomyces* strains are selectively interacting with the wheat cultivars especially in saline conditions. Heliyon 6, e03445. doi: 10.1016/j.heliyon.2020.e03445 32095655 PMC7033526

[B3] Alavi P. Starcher M. R. Zachow C. Müller H. Berg G. (2013). Root-microbe systems: The effect and mode of interaction of stress protecting agent (SPA) *Stenotrophomonas rhizophila* DSM14405T. Front. Plant Sci. 4. doi: 10.3389/fpls.2013.00141 PMC3653106 23717321

[B4] Amaresan N. Jayakumar V. Kumar K. Thajuddin N. (2019). Biocontrol and plant growth-promoting ability of plant-associated bacteria from tomato (*Lycopersicum esculentum*) under field condition. Microb. Pathog. 136, 103713. doi: 10.1016/j.micpath.2019.103713 31491553

[B5] Asari S. Tarkowská D. Rolčík J. Novák O. Palmero D. V. Bejai S.. (2017). Analysis of plant growth-promoting properties of *Bacillus amyloliquefaciens* UCMB5113 using *Arabidopsis thaliana* as host plant. Planta 245, 15–30. doi: 10.1007/s00425-016-2580-9 27541497 PMC5226999

[B6] Badri D. V. Vivanco J. M. (2009). Regulation and function of root exudates. Plant Cell Environ. 32, 666–681. doi: 10.1111/j.1365-3040.2009.01926.x 19143988

[B7] Bais H. P. Weir T. L. Perry L. G. Gilroy S. Vivanco J. M. (2006). The role of root exudates in rhizosphere interactions with plants and other organisms. Annu. Rev. Plant Biol. 57, 233–266. doi: 10.1146/annurev.arplant.57.032905.105159 16669762

[B8] Ben Abdallah D. Frikha-Gargouri O. Tounsi S. (2018). Rizhospheric competence, plant growth promotion and biocontrol efficacy of *Bacillus amyloliquefaciens* subsp. *plantarum* strain 32a. Biol. Control 124, 61–67. doi: 10.1016/j.biocontrol.2018.01.013

[B9] Berg G. (2009). Plant-microbe interactions promoting plant growth and health: Perspectives for controlled use of microorganisms in agriculture. Appl. Microbiol. Biotechnol. 84, 11–18. doi: 10.1007/s00253-009-2092-7 19568745

[B10] Blandino M. Battisti M. Vanara F. Reyneri A. (2022). The synergistic effect of nitrogen and phosphorus starter fertilization sub-surface banded at sowing on the early vigor, grain yield and quality of maize. Eur. J. Agron. 137, 126509. doi: 10.1016/j.eja.2022.126509

[B11] Blandino M. Scarpino V. Giordano D. Sulyok M. Krska R. Vanara F.. (2017). Impact of sowing time, hybrid and environmental conditions on the contamination of maize by emerging mycotoxins and fungal metabolites. Ital. J. Agron. 12, 928. doi: 10.4081/ija.2017.928

[B12] Canarini A. Kaiser C. Merchant A. Richter A. Wanek W. (2019). Root exudation of primary metabolites: Mechanisms and their roles in plant responses to environmental stimuli. Front. Plant Sci. 10. doi: 10.3389/fpls.2019.00157 PMC6407669 30881364

[B13] Carpenter S. R. Caraco N. F. Correll D. L. Howarth R. W. Sharpley A. N. Smith V. H. (1998). Nonpoint pollution of surface waters with phosphorus and nitrogen. Ecol. Appl. 8, 559–568. doi: 10.1890/1051-0761(1998)008[0559:NPOSWW]2.0.CO;2

[B14] Chabot R. Antoun H. Cescas M. P. (1996). Growth promotion of maize and lettuce by phosphate-solubilizing *Rhizobium leguminosarum* biovar. phaseoli. Plant Soil 184, 311–321. doi: 10.1007/BF00010460

[B15] Chandra D. Srivastava R. Gupta V. V. S. R. Franco C. M. M. Paasricha N. Saifi S. K.. (2019). Field performance of bacterial inoculants to alleviate water stress effects in wheat (*Triticum aestivum* L.). Plant Soil 441, 261–281. doi: 10.1007/s11104-019-04115-9

[B16] Chen L. Li K. Shang J. Wu Y. Chen T. Wanyan Y.. (2021). Plant growth-promoting bacteria improve maize growth through reshaping the rhizobacterial community in low-nitrogen and low-phosphorus soil. Biol. Fertil. Soils 57, 1075–1088. doi: 10.1007/s00374-021-01598-6

[B17] Chen S. Waghmode T. R. Sun R. Kuramae E. E. Hu C. Liu B. (2019). Root-associated microbiomes of wheat under the combined effect of plant development and nitrogen fertilization. Microbiome 7, 136. doi: 10.1186/s40168-019-0750-2 31640813 PMC6806522

[B18] Chen X.-H. Koumoutsi A. Scholz R. Borriss R. (2009). More than anticipated – Production of antibiotics and other secondary metabolites by *Bacillus amyloliquefaciens* FZB42. J. Mol. Microbiol. Biotechnol. 16, 14–24. doi: 10.1159/000142891 18957859

[B19] Chowdhury S. P. Dietel K. Rändler M. Schmid M. Junge H. Borriss R.. (2013). Effects of *Bacillus amyloliquefaciens* FZB42 on lettuce growth and health under pathogen pressure and its impact on the rhizosphere bacterial community. PloS One 8, e68818. doi: 10.1371/journal.pone.0068818 23935892 PMC3720850

[B20] Correa O. S. Montecchia M. S. Berti M. F. Fernández Ferrari M. C. Pucheu N. L. Kerber N. L.. (2009). *Bacillus amyloliquefaciens* BNM122, a potential microbial biocontrol agent applied on soybean seeds, causes a minor impact on rhizosphere and soil microbial communities. Appl. Soil Ecol. 41, 185–194. doi: 10.1016/j.apsoil.2008.10.007

[B21] Correa-Galeote D. Bedmar E. J. Fernández-González A. J. Fernández-López M. Arone G. J. (2016). Bacterial communities in the rhizosphere of amilaceous maize (*Zea mays* L.) as assessed by pyrosequencing. Front. Plant Sci. 7. doi: 10.3389/fpls.2016.01016 PMC4966391 27524985

[B22] Cozzolino V. Monda H. Savy D. Di Meo V. Vinci G. Smalla K. (2021). Cooperation among phosphate-solubilizing bacteria, humic acids and arbuscular mycorrhizal fungi induces soil microbiome shifts and enhances plant nutrient uptake. Chem. Biol. Technol. Agric. 8, 31. doi: 10.1186/s40538-021-00230-x

[B23] Dal Cortivo C. Ferrari M. Visioli G. Lauro M. Fornasier F. Barion G.. (2020). Effects of seed-applied biofertilizers on rhizosphere biodiversity and growth of common wheat (*Triticum aestivum* L.) in the field. Front. Plant Sci. 11. doi: 10.3389/fpls.2020.00072 PMC7054350 32174929

[B24] Damo J. L. C. Ramirez M. D. A. Agake S.-I. Pedro M. Brown M. Sekimoto H.. (2022). Isolation and characterization of phosphate solubilizing bacteria from paddy field soils in Japan. Microb. Environ. 37, ME21085. doi: 10.1264/jsme2.ME21085 PMC9530731 35598988

[B25] de Oliveira A. L. M. de Canuto E. L. Urquiaga S. Reis V. M. Baldini J. I. (2006). Yield of micropropagated sugarcane varieties in different soil types following inoculation with diazotrophic bacteria. Plant Soil 284, 23–32. doi: 10.1007/s11104-006-0025-0

[B26] Edgar R. C. (2004a). MUSCLE: A multiple sequence alignment method with reduced time and space complexity. BMC Bioinform. 5, 113. doi: 10.1186/1471-2105-5-113 PMC517706 15318951

[B27] Edgar R. C. (2004b). MUSCLE: Multiple sequence alignment with high accuracy and high throughput. Nucleic Acids Res. 32, 1792–1797. doi: 10.1093/nar/gkh340 15034147 PMC390337

[B28] Eltlbany N. Baklawa M. Ding G.-C. Nassal D. Weber N. Kandeler E.. (2019). Enhanced tomato plant growth in soil under reduced P supply through microbial inoculants and microbiome shifts. FEMS Microbiol. Ecol. 95, fiz124. doi: 10.1093/femsec/fiz124 31386159

[B29] Ercole T. G. Savi D. C. Adamoski D. Kava V. M. Hungria M. Galli-Terasawa L. V. (2021). Diversity of maize (*Zea mays* L.) rhizobacteria with potential to promote plant growth. Braz. J. Microbiol. 52, 1807–1823. doi: 10.1007/s42770-021-00596-y 34458975 PMC8578223

[B30] FAOSTAT (2022). Crops and livestock products database (Rome, Italy: Food and Agriculture Organization of United Nations). Available at: https://www.fao.org/faostat/en/#data/QCL.

[B31] Favela A. Bohn M. O. Kent A. D. (2021). Maize germplasm chronosequence shows crop breeding history impacts recruitment of the rhizosphere microbiome. ISME J. 15, 2454–2464. doi: 10.1038/s41396-021-00923-z 33692487 PMC8319409

[B32] García-Lara S. Serna-Saldivar S. O. (2019). ““Corn history and culture”,” in Corn, 3rd. Ed. Serna-Saldivar S. O. (Oxford, UK: Woodhead Publishing and AACC International Press), 1–18.

[B33] García-Salamanca A. Molina-Henares M. A. van Dillewijn P. Solano J. Pizarro-Tobías P. Roca A.. (2013). Bacterial diversity in the rhizosphere of maize and the surrounding carbonate-rich bulk soil. Microb. Biotechnol. 6, 36–44. doi: 10.1111/j.1751-7915.2012.00358.x 22883414 PMC3815383

[B34] Ghavami N. Alikhani H. A. Pourbabaei A. A. Besharati H. (2017). Effects of two new siderophore-producing rhizobacteria on growth and iron content of maize and canola plants. J. Plant Nutr. 40, 736–746. doi: 10.1080/01904167.2016.1262409

[B35] Gomes E. A. Lana U. G. P. Quensen J. F. de Sousa S. M. Oliveira C. A. Guimarães L. J. M.. (2018). Root-associated microbiome of maize genotypes with contrasting phosphorus use efficiency. Phytobiomes J. 2, 129–137. doi: 10.1094/PBIOMES-03-18-0012-R

[B36] Gómez Expósito R. Postma J. Raaijmakers J. M. De Bruijn I. (2015). Diversity and activity of *Lysobacter* species from disease suppressive soils. Front. Microbiol. 6. doi: 10.3389/fmicb.2015.01243 PMC4644931 26635735

[B37] Gontia-Mishra I. Sapre S. Kachare S. Tiwari S. (2017). Molecular diversity of 1-aminocyclopropane-1-carboxylate (ACC) deaminase producing PGPR from wheat (*Triticum aestivum* L.) rhizosphere. Plant Soil 414, 213–227. doi: 10.1007/s11104-016-3119-3

[B38] Gontia-Mishra I. Sapre S. Sharma A. Tiwari S. (2016). Amelioration of drought tolerance in wheat by the interaction of plant growth-promoting rhizobacteria. Plant Biol. 18, 992–1000. doi: 10.1111/plb.12505 27607023

[B39] Gordon N. S. Valenzuela A. Adams S. M. Ramsey P. W. Pollock J. L. Holben W. E.. (2009). *Pedobacter nyackensis* sp. nov., Pedobacter alluvionis sp. nov. and *Pedobacter borealis* sp. nov., isolated from Montana flood-plain sediment and forest soil. Int. J. Syst. Evol. Microbiol. 59, 1720–1726. doi: 10.1099/ijs.0.000158-0 19542109

[B40] Gouda S. Kerry R. G. Das G. Paramithiotis S. Shin H.-S. Patra J. K. (2018). Revitalization of plant growth promoting rhizobacteria for sustainable development in agriculture. Microbiol. Res. 206, 131–140. doi: 10.1016/j.micres.2017.08.016 29146250

[B41] Guo J. H. Liu X. J. Zhang Y. Shen J. L. Han W. X. Zhang W. F.. (2010). Significant acidification in major Chinese croplands. Science 327, 1008–1010. doi: 10.1126/science.1182570 20150447

[B42] Guo L. Wang C. Shen R. F. (2022). Stronger effects of maize rhizosphere than phosphorus fertilization on phosphatase activity and phosphorus-mineralizing-related bacteria in acidic soils. Rhizosphere 23, 100555. doi: 10.1016/j.rhisph.2022.100555

[B43] Hameeda B. Rupela O. P. Reddy G. Satyavani K. (2006). Application of plant growth-promoting bacteria associated with composts and macrofauna for growth promotion of pearl millet (*Pennisetum glaucum* L.). Biol. Fertil. Soils 43, 221–227. doi: 10.1007/s00374-006-0098-1

[B44] Han L. Wang Z. Li N. Wang Y. Feng J. Zhang X. (2019). *Bacillus amyloliquefaciens* B1408 suppresses *Fusarium* wilt in cucumber by regulating the rhizosphere microbial community. Appl. Soil Ecol. 136, 55–66. doi: 10.1016/j.apsoil.2018.12.011

[B45] Hayat R. Ali S. Amara U. Khalid R. Ahmed I. (2010). Soil beneficial bacteria and their role in plant growth promotion: A review. Ann. Microbiol. 60, 579–598. doi: 10.1007/s13213-010-0117-1

[B46] Hayward A. C. Fegan N. Fegan M. Stirling G. R. (2010). *Stenotrophomonas* and *Lysobacter*: Ubiquitous plant-associated gamma-proteobacteria of developing significance in applied microbiology. J. Appl. Microbiol. 108, 756–770. doi: 10.1111/j.1365-2672.2009.04471.x 19702860

[B47] Higdon S. M. Pozzo T. Tibbett E. J. Chiu C. Jeannotte R. Weimer B. C.. (2020). Diazotrophic bacteria from maize exhibit multifaceted plant growth promotion traits in multiple hosts. PloS One 15, e0239081. doi: 10.1371/journal.pone.0239081 32925972 PMC7489573

[B48] Hrynkiewicz K. Baum C. Leinweber P. (2010). Density, metabolic activity, and identity of cultivable rhizosphere bacteria on *Salix viminalis* in disturbed arable and landfill soils. J. Plant Nutr. Soil Sci. 173, 747–756. doi: 10.1002/jpln.200900286

[B49] Hu L. Robert C. A. M. Cadot S. Zhang X. Ye M. Li B.. (2018). Root exudate metabolites drive plant-soil feedbacks on growth and defense by shaping the rhizosphere microbiota. Nat. Commun. 9, 2738. doi: 10.1038/s41467-018-05122-7 30013066 PMC6048113

[B50] Hui C. Sun P. Guo X. Jiang H. Zhao Y. Xu L. (2018). Shifts in microbial community structure and soil nitrogen mineralization following short-term soil amendment with the ammonifier *Bacillus amyloliquefaciens* DT. Int. Biodeterior. Biodegrad. 132, 40–48. doi: 10.1016/j.ibiod.2018.05.008

[B51] Hungria M. Campo R. J. Souza E. M. Pedrosa F. O. (2010). Inoculation with selected strains of *Azospirillum brasilense* and A. lipoferum improves yields of maize and wheat in Brazil. Plant Soil 331, 413–425. doi: 10.1007/s11104-009-0262-0

[B52] Idris E. E. Iglesias D. J. Talon M. Borriss R. (2007). Tryptophan-dependent production of indole-3-acetic acid (IAA) affects level of plant growth promotion by *Bacillus amyloliquefaciens* FZB42. Mol. Plant Microbe Interact. 20, 619–626. doi: 10.1094/MPMI-20-6-0619 17555270

[B53] Idriss E. E. Makarewicz O. Farouk A. Rosner K. Greiner R. Bochow H.. (2002). Extracellular phytase activity of *Bacillus amyloliquefaciens* FZB45 contributes to its plant-growth-promoting effect. Microbiology 148, 2097–2109. doi: 10.1099/00221287-148-7-2097 12101298

[B54] Ikeda A. C. Bassani L. L. Adamoski D. Stringari D. Cordeiro V. K. Glienke C.. (2013). Morphological and genetic characterization of endophytic bacteria isolated from roots of different maize genotypes. Microb. Ecol. 65, 154–160. doi: 10.1007/s00248-012-0104-0 22956211

[B55] Imran M. Mahmood A. Römheld V. Neumann G. (2013). Nutrient seed priming improves seedling development of maize exposed to low root zone temperatures during early growth. Eur. J. Agron. 49, 141–148. doi: 10.1016/j.eja.2013.04.001

[B56] Juo A. S. R. Dabiri A. Franzluebbers K. (1995). Acidification of a kaolinitic Alfisol under continuous cropping with nitrogen fertilization in West Africa. Plant Soil 171, 245–253. doi: 10.1007/BF00010278

[B57] Kaiser D. E. Coulter J. A. Vetsch J. A. (2016). Maize hybrid response to in-furrow starter fertilizer as affected by planting date. Agron. J. 108, 2493–2501. doi: 10.2134/agronj2016.02.0124

[B58] Kavino M. Harish S. Kumar N. Saravanakumar D. Samiyappan R. (2010). Effect of chitinolytic PGPR on growth, yield and physiological attributes of banana (*Musa* spp.) under field conditions. Appl. Soil Ecol. 45, 71–77. doi: 10.1016/j.apsoil.2010.02.003

[B59] Kerstens M. Hesen V. Yalamanchili K. Bimbo A. Grigg S. Opdenacker D.. (2021). Nature and nurture: Genotype-dependent differential responses of root architecture to agar and soil environments. Genes 12, 1028. doi: 10.3390/genes12071028 34356045 PMC8303133

[B60] Kimura M. (1980). A simple method for estimating evolutionary rates of base substitutions through comparative studies of nucleotide sequences. J. Mol. Evol. 16, 111–120. doi: 10.1007/BF01731581 7463489

[B61] Kostka J. E. Green S. J. Rishishwar L. Prakash O. Katz L. S. Mariño-Ramírez L.. (2012). Genome sequences for six *Rhodanobacter* strains, isolated from soils and the terrestrial subsurface, with variable denitrification capabilities. J. Bacteriol. 194, 4461–4462. doi: 10.1128/JB.00871-12 22843592 PMC3416251

[B62] Kröber M. Wibberg D. Grosch R. Eikmeyer F. Verwaaijen B. Chowdhury S. P.. (2014). Effect of the strain *Bacillus amyloliquefaciens* FZB42 on the microbial community in the rhizosphere of lettuce under field conditions analyzed by whole metagenome sequencing. Front. Microbiol. 5. doi: 10.3389/fmicb.2014.00252 PMC4033844 24904564

[B63] Lazcano C. Gómez-Brandón M. Revilla P. Domínguez J. (2013). Short-term effects of organic and inorganic fertilizers on soil microbial community structure and function: A field study with sweet maize. Biol. Fertil. Soils 49, 723–733. doi: 10.1007/s00374-012-0761-7

[B64] Li C. Cao P. Du C. Zhang X. Bing H. Li L.. (2021). *Massilia rhizosphaerae* sp. nov., a rice-associated rhizobacterium with antibacterial activity against *Ralstonia solanacearum* . Int. J. Syst. Evol. Microbiol. 71, 5009. doi: 10.1099/ijsem.0.005009 34520338

[B65] Li X. Rui J. Mao Y. Yannarell A. Mackie R. (2014). Dynamics of the bacterial community structure in the rhizosphere of a maize cultivar. Soil Biol. Biochem. 68, 392–401. doi: 10.1016/j.soilbio.2013.10.017

[B66] Long N. V. Assefa Y. Schwalbert R. Ciampitti I. A. (2017). Maize yield and planting date relationship: A synthesis-analysis for US high-yielding contest-winner and field research data. Front. Plant Sci. 8. doi: 10.3389/fpls.2017.02106 PMC5743010 29312377

[B67] Lu Y. Wassmann R. Neue H. U. Huang C. (1999). Impact of phosphorus supply on root exudation, aerenchyma formation and methane emission of rice plants. Biogeochemistry 47, 203–218. doi: 10.1007/BF00994923

[B68] Luo L. Zhao C. Wang E. Raza A. Yin C. (2022). *Bacillus amyloliquefaciens* as an excellent agent for biofertilizer and biocontrol in agriculture: An overview for its mechanisms. Microbiol. Res. 259, 127016. doi: 10.1016/j.micres.2022.127016 35390741

[B69] Ma Q. Wang X. Li H. Li H. Zhang F. Rengel Z.. (2015). Comparing localized application of different N fertilizer species on maize grain yield and agronomic N-use efficiency on a calcareous soil. Field Crops Res. 180, 72–79. doi: 10.1016/j.fcr.2015.05.011

[B70] Maarastawi S. A. Frindte K. Geer R. Kröber E. Knief C. (2018). Temporal dynamics and compartment specific rice straw degradation in bulk soil and the rhizosphere of maize. Soil Biol. Biochem. 127, 200–212. doi: 10.1016/j.soilbio.2018.09.028

[B71] Manter D. K. Delgado J. A. Holm D. G. Strong R. A. (2010). Pyrosequencing reveals a highly diverse and cultivar-specific bacterial endophyte community in potato roots. Microb. Ecol. 60, 157–166. doi: 10.1007/s00248-010-9658-x 20414647

[B72] Matsuyama N. Saigusa M. Sakaiya E. Tamakawa K. Oyamada Z. Kudo K. (2005). Acidification and soil productivity of allophanic Andosols affected by heavy application of fertilizers. Soil Sci. Plant Nutr. 51, 117–123. doi: 10.1111/j.1747-0765.2005.tb00014.x

[B73] Medina-de la Rosa G. López-Reyes L. Carcaño-Montiel M. G. López-Olguín J. F. Hernández-Espinosa M.Á. Rivera-Tapia J. A. (2016). Rhizosphere bacteria of maize with chitinolytic activity and its potential in the control of phytopathogenic fungi. Arch. Phytopathol. Plant Prot. 49, 310–321. doi: 10.1080/03235408.2016.1201345

[B74] Mendes R. Garbeva P. Raaijmakers J. M. (2013). The rhizosphere microbiome: Significance of plant beneficial, plant pathogenic, and human pathogenic microorganisms. FEMS Microbiol. Rev. 37, 634–663. doi: 10.1111/1574-6976.12028 23790204

[B75] Miranda-Carrazco A. Navarro-Noya Y. E. Govaerts B. Verhulst N. Dendooven L. (2022). Nitrogen fertilizer application alters the root endophyte bacterial microbiome in maize plants, but not in the stem or rhizosphere soil. Microbiol. Spectr. 10, e01785–e01722. doi: 10.1128/spectrum.01785-22 36255324 PMC9769722

[B76] Mishra A. Chauhan P. S. Chaudhry V. Tripathi M. Nautiyal C. S. (2011). Rhizosphere competent *Pantoea agglomerans* enhances maize (*Zea mays*) and chickpea (*Cicer arietinum* L.) growth, without altering the rhizosphere functional diversity. Antonie van Leeuwenhoek 100, 405–413. doi: 10.1007/s10482-011-9596-8 21638110

[B77] Nishioka T. Marian M. Kobayashi I. Kobayashi Y. Yamamoto K. Tamaki H.. (2019). Microbial basis of *Fusarium* wilt suppression by *Allium* cultivation. Sci. Rep. 9, 1715. doi: 10.1038/s41598-018-37559-7 30737419 PMC6368641

[B78] Palla M. Turrini A. Cristani C. Bonora L. Pellegrini D. Primicerio J.. (2022). Impact of sheep wool residues as soil amendments on olive beneficial symbionts and bacterial diversity. Bioresour. Bioprocess. 9, 45. doi: 10.1186/s40643-022-00534-2 PMC10992544 38647844

[B79] Peiffer J. A. Spor A. Koren O. Jin Z. Tringe S. G. Dangl J. L.. (2013). Diversity and heritability of the maize rhizosphere microbiome under field conditions. Proc. Natl. Acad. Sci. U.S.A. 110, 6548–6553. doi: 10.1073/pnas.1302837110 23576752 PMC3631645

[B80] Pérez-Jaramillo J. E. Carrión V. J. Bosse M. Ferrão L. F. V. de Hollander M. Garcia A. A. F.. (2017). Linking rhizosphere microbiome composition of wild and domesticated *Phaseolus vulgaris* to genotypic and root phenotypic traits. ISME J. 11, 2244–2257. doi: 10.1038/ismej.2017.85 28585939 PMC5607367

[B81] Peter R. Eschholz T. W. Stamp P. Liedgens M. (2009). Early growth of flint maize landraces under cool conditions. Crop Sci. 49, 169–178. doi: 10.2135/cropsci2007.10.0538

[B82] Philippot L. Raaijmakers J. M. Lemanceau P. van der Putten W. H. (2013). Going back to the roots: The microbial ecology of the rhizosphere. Nat. Rev. Microbiol. 11, 789–799. doi: 10.1038/nrmicro3109 24056930

[B83] Puopolo G. Tomada S. Pertot I. (2018). The impact of the omics era on the knowledge and use of *Lysobacter* species to control phytopathogenic microorganisms. J. Appl. Microbiol. 124, 15–27. doi: 10.1111/jam.13607 28992371

[B84] Qaisrani M. M. Zaheer A. Mirza M. S. Naqqash T. Qaisrani T. B. Hanif M. K.. (2019). A comparative study of bacterial diversity based on culturable and culture-independent techniques in the rhizosphere of maize (*Zea mays* L.). Saudi J. Biol. Sci. 26, 1344–1351. doi: 10.1016/j.sjbs.2019.03.010 31762594 PMC6864194

[B85] Qiao J.-Q. Wu H.-J. Huo R. Gao X.-W. Borriss R. (2014). Stimulation of plant growth and biocontrol by *Bacillus amyloliquefaciens* subsp. *plantarum* FZB42 engineered for improved action. Chem. Biol. Technol. Agric. 1, 12. doi: 10.1186/s40538-014-0012-2

[B86] Qin Y. Shang Q. Zhang Y. Li P. Chai Y. (2017). *Bacillus amyloliquefaciens* L-S60 reforms the rhizosphere bacterial community and improves growth conditions in cucumber plug seedling. Front. Microbiol. 8. doi: 10.3389/fmicb.2017.02620 PMC5744474 29312278

[B87] Rahman M. Sabir A. A. Mukta J. A. Khan M. M. A. Mohi-Ud-Din M. Miah M. G.. (2018). Plant probiotic bacteria *Bacillus* and *Paraburkholderia* improve growth, yield and content of antioxidants in strawberry fruit. Sci. Rep. 8, 2504. doi: 10.1038/s41598-018-20235-1 29410436 PMC5802727

[B88] Raths R. Peta V. Bücking H. (2020). *Massilia arenosa* sp. nov., isolated from the soil of a cultivated maize field. Int. J. Syst. Evol. Microbiol. 70, 3912–3920. doi: 10.1099/ijsem.0.004266 32511088

[B89] Reis V. U. V. Penido A. C. Carvalho E. R. Rocha D. K. Reis L. V. Semolini P. H. Z. (2022). Vigor of maize seeds and its effects on plant stand establishment, crop development and grain yield. J. Seed Sci. 44, e202244020. doi: 10.1590/2317-1545v44257527

[B90] Robertson G. P. Vitousek P. M. (2009). Nitrogen in agriculture: Balancing the cost of an essential resource. Annu. Rev. Environ. Resour. 34, 97–125. doi: 10.1146/annurev.environ.032108.105046

[B91] Roesch L. F. W. Fulthorpe R. R. Riva A. Casella G. Hadwin A. K. M. Kent A. D.. (2007). Pyrosequencing enumerates and contrasts soil microbial diversity. ISME J. 1, 283–290. doi: 10.1038/ismej.2007.53 18043639 PMC2970868

[B92] Rouphael Y. Colla G. (2020). Editorial: biostimulants in agriculture. Front. Plant Sci. 11. doi: 10.3389/fpls.2020.00040 PMC7010726 32117379

[B93] Ruzzi M. Aroca R. (2015). Plant growth-promoting rhizobacteria act as biostimulants in horticulture. Scientia Hortic. 196, 124–134. doi: 10.1016/j.scienta.2015.08.042

[B94] Ryan R. P. Monchy S. Cardinale M. Taghavi S. Crossman L. Avison M. B.. (2009). The versatility and adaptation of bacteria from the genus *Stenotrophomonas.* Nat. Rev. Microbiol. 7, 514–525. doi: 10.1038/nrmicro2163 19528958

[B95] Sang M. K. Kim K. D. (2012). The volatile-producing *Flavobacterium johnsoniae* strain GSE09 shows biocontrol activity against *Phytophthora capsici* in pepper. J. Appl. Microbiol. 113, 383–398. doi: 10.1111/j.1365-2672.2012.05330.x 22563881

[B96] Sasse J. Martinoia E. Northern T. (2018). Feed your friends: Do plant exudates shape the root microbiome? Trends Plant Sci. 23, 25–41. doi: 10.1016/j.tplants.2017.09.003 29050989

[B97] Schröder J. J. Vermeulen G. D. van der Schoot J. R. van Dijk W. Huijsmans J. F. M. Meuffels G. J. H. M.. (2015). Maize yields benefit from injected manure positioned in bands. Eur. J. Agron. 64, 29–36. doi: 10.1016/j.eja.2014.12.011

[B98] Semchenko M. Xue P. Leigh T. (2021). Functional diversity and identity of plant genotypes regulate rhizodeposition and soil microbial activity. New Phytol. 232, 776–787. doi: 10.1111/nph.17604 34235741

[B99] Semenov M. V. Krasnov G. S. Semenov V. M. van Bruggen A. H. C. (2020). Long-term fertilization rather than plant species shapes rhizosphere and bulk soil prokaryotic communities in agroecosystems. Appl. Soil Ecol. 154, 103641. doi: 10.1016/j.apsoil.2020.103641

[B100] Sharma M. Sood G. Chauhan A. (2021). Bioprospecting beneficial endophytic bacterial communities associated with *Rosmarinus officinalis* for sustaining plant health and productivity. World J. Microbiol. Biotechnol. 37, 135. doi: 10.1007/s11274-021-03101-7 34263378

[B101] Shen M. Li J. Dong Y. Zhang Z. Zhao Y. Li Q.. (2021). The effects of microbial inoculants on bacterial communities of the rhizosphere soil of maize. Agriculture 11, 389. doi: 10.3390/agriculture11050389

[B102] Shen Z. Ruan Y. Chao X. Zhang J. Li R. Shen Q. (2015). Rhizosphere microbial community manipulated by 2 years of consecutive biofertilizer application associated with banana *Fusarium* wilt disease suppression. Biol. Fertil. Soils 51, 553–562. doi: 10.1007/s00374-015-1002-7

[B103] Shenton M. Iwamoto C. Kurata N. Ikeo K. (2016). Effect of wild and cultivated rice genotypes on rhizosphere bacterial community composition. Rice 9, 42. doi: 10.1186/s12284-016-0111-8 27557607 PMC4996804

[B104] Shrestha A. Kim B. S. Park D. H. (2014). Biological control of bacterial spot disease and plant growth-promoting effects of lactic acid bacteria on pepper. Biocontrol Sci. Technol. 24, 763–779. doi: 10.1080/09583157.2014.894495

[B105] Silva U. C. Medeiros J. D. Leite L. R. Morais D. K. Cuadros-Orellana S. Oliveira C. A.. (2017). Long-term rock phosphate fertilization impacts the microbial communities of maize rhizosphere. Front. Microbiol. 8. doi: 10.3389/fmicb.2017.01266 PMC5504191 28744264

[B106] Singh R. P. Jha P. N. (2017). The PGPR Stenotrophomonas maltophilia SBP-9 augments resistance against biotic and abiotic stress in wheat plants. Front. Microbiol. 8. doi: 10.3389/fmicb.2017.01945 PMC5640710 29062306

[B107] Soil Survey Staff (2010). Keys to soil taxonomy. 11th ed (Washington, DC, USA: USDA Natural Resources Conservation Service).

[B108] Steyn P. L. Segers P. Vancanneyt M. Sandra P. Kerstens K. Joubert J. J. (1998). Classification of heparinolytic bacteria into a new genus, *Pedobacter*, comprising four species: *Pedobacter heparinus* comb. nov., *Pedobacter piscium* comb. nov., Pedobacter africanus sp. nov. and *Pedobacter saltans* sp. nov. Proposal of the family Sphingobacteriaceae fam. nov. Int. J. Syst. Bacteriol 48, 165–177. doi: 10.1099/00207713-48-1-165 9542086

[B109] Sun R. Zhang X.-X. Guo X. Wang D. Chu H. (2015). Bacterial diversity in soils subjected to long-term chemical fertilization can be more stably maintained with the addition of livestock manure than wheat straw. Soil Biol. Biochem. 88, 9–18. doi: 10.1016/j.soilbio.2015.05.007

[B110] Tamura K. Stecher G. Kumar S. (2021). MEGA11: Molecular evolutionary genetics analysis version 11. Mol. Biol. Evol. 38, 3022–3027. doi: 10.1093/molbev/msab120 33892491 PMC8233496

[B111] Tang X. Placella S. A. Daydé F. Bernard L. Robin A. Journet E.-P.. (2016). Phosphorus availability and microbial community in the rhizosphere of intercropped cereal and legume along a P-fertilizer gradient. Plant Soil 407, 119–134. doi: 10.1007/s11104-016-2949-3

[B112] Tiwari S. Prasad V. Chauhan P. S. Lata C. (2017). *Bacillus amyloliquefaciens* confers tolerance to various abiotic stresses and modulates plant response to phytohormones through osmoprotection and gene expression regulation in rice. Front. Plant Sci. 8. doi: 10.3389/fpls.2017.01510 PMC5581838 28900441

[B113] Tucci M. Ruocco M. De Masi L. De Palma M. Lorito M. (2011). The beneficial effect of Trichoderma spp. on tomato is modulated by the plant genotype. Mol. Plant Pathol. 12, 341–354. doi: 10.1111/j.1364-3703.2010.00674.x 21453429 PMC6640367

[B114] Turnbull A. L. Liu Y. Lazarovits G. (2012). Isolation of bacteria from the rhizosphere and rhizoplane of potato (*Solanum tuberosum*) grown in two distinct soils using semi selective media and characterization of their biological properties. Am. J. Potato Res. 89, 294–305. doi: 10.1007/s12230-012-9253-4

[B115] Turrini A. Agnolucci M. Palla M. Tomé E. Tagliavini M. Scandellari F.. (2017). Species diversity and community composition of native arbuscular mycorrhizal fungi in apple roots are affected by site and orchard management. Appl. Soil Ecol. 116, 42–54. doi: 10.1016/j.apsoil.2017.03.016

[B116] Ujvári G. Turrini A. Avio L. Agnolucci M. (2021). Possible role of arbuscular mycorrhizal fungi and associated bacteria in the recruitment of endophytic bacterial communities by plant roots. Mycorrhiza 31, 527–544. doi: 10.1007/s00572-021-01040-7 34286366 PMC8484141

[B117] van den Heuvel R. N. van der Biezen E. Jetten M. S. M. Hefting M. M. Kartal B. (2010). Denitrification at pH 4 by a soil-derived *Rhodanobacter*-dominated community. Environ. Microbiol. 12, 3264–3271. doi: 10.1111/j.1462-2920.2010.02301.x 20649643

[B118] van der Heijden M. G. A. Bardgett R. D. van Straalen N. M. (2008). The unseen majority: Soil microbes as drivers of plant diversity and productivity in terrestrial ecosystems. Ecol. Lett. 11, 296–310. doi: 10.1111/j.1461-0248.2007.01139.x 18047587

[B119] Velásquez A. Vega-Celedón P. Fiaschi G. Agnolucci M. Avio L. Giovannetti M.. (2020). Responses of *Vitis vinifera* cv. Cabernet Sauvignon roots to the arbuscular mycorrhizal fungus *Funneliformis mosseae* and the plant growth-promoting rhizobacterium *Ensifer meliloti* include changes in volatile organic compounds. Mycorrhiza 30, 161–170. doi: 10.1007/s00572-020-00933-3 31974639

[B120] Walters W. A. Jin Z. Youngblut N. Wallace J. G. Sutter J. Zhang W.. (2018). Large-scale replicated field study of maize rhizosphere identifies heritable microbes. Proc. Natl. Acad. Sci. U.S.A. 115, 7368–7373. doi: 10.1073/pnas.1800918115 29941552 PMC6048482

[B121] Wang Q. Jiang X. Guan D. Wei D. Zhao B. Ma M.. (2018). Long-term fertilization changes bacterial diversity and bacterial communities in the maize rhizosphere of Chinese Mollisols. Appl. Soil Ecol. 125, 88–96. doi: 10.1016/j.apsoil.2017.12.007

[B122] Wang B. Shen Z. Zhang F. Raza W. Yuan J. Huang R.. (2016). *Bacillus amyloliquefaciens* strain W19 can promote growth and yield and suppress *Fusarium* wilt in banana under greenhouse and field conditions. Pedosphere 26, 733–744. doi: 10.1016/S1002-0160(15)60083-2

[B123] Waqas M. A. Wang X. Zafar S. A. Noor M. A. Hussain H. A. Azher Nawaz M.. (2021). Thermal stresses in maize: Effects and management strategies. Plants 10, 293. doi: 10.3390/plants10020293 33557079 PMC7913793

[B124] Wen X. Wang M. Ti J. Wu Y. Chen F. (2017). Bacterial community composition in the rhizosphere of maize cultivars widely grown in different decades. Biol. Fertil. Soils 53, 221–229. doi: 10.1007/s00374-016-1169-6

[B125] Withers P. J. A. Haygarth P. M. (2007). Agriculture, phosphorus and eutrophication: A European perspective. Soil Use Manage. 23, 1–4. doi: 10.1111/j.1475-2743.2007.00116.x

[B126] Wu Y. Zhou J. Li C. Ma Y. (2019). Antifungal and plant growth promotion activity of volatile organic compounds produced by *Bacillus amyloliquefaciens* . MicrobiologyOpen 8, e813. doi: 10.1002/mbo3.813 PMC6692555 30907064

[B127] Yang Y. Wang N. Guo X. Zhang Y. Ye B. Luo Y. (2017). Comparative analysis of bacterial community structure in the rhizosphere of maize by high-throughput pyrosequencing. PloS One 12, e0178425. doi: 10.1371/journal.pone.0178425 28542542 PMC5444823

[B128] Yin C. Casa Vargas J. M. Schlatter D. C. Hagerty C. H. Hulbert S. H. Paulitz T. C. (2021). Rhizosphere community selection reveals bacteria associated with reduced root disease. Microbiome 9, 86. doi: 10.1186/s40168-020-00997-5 33836842 PMC8035742

[B129] Yoon J.-H. Kang S.-J. Park S. Oh T.-K. (2007). *Pedobacter lentus* sp. nov. and Pedobacter terricola sp. nov., isolated from soil. Int. J. Syst. Evol. Microbiol. 57, 2089–2095. doi: 10.1099/ijs.0.65146-0 17766877

[B130] Youseif S. H. (2018). Genetic diversity of plant growth promoting rhizobacteria and their effects on the growth of maize plants under greenhouse conditions. Ann. Agric. Sci. 63, 25–35. doi: 10.1016/j.aoas.2018.04.002

[B131] Yu X. Liu X. Zhu T. H. Liu G. H. Mao C. (2011). Isolation and characterization of phosphate-solubilizing bacteria from walnut and their effect on growth and phosphorus mobilization. Biol. Fertil. Soils 47, 437–446. doi: 10.1007/s00374-011-0548-2

[B132] Yu Z. Morrison M. (2004). Comparisons of different hypervariable regions of *rrs* genes for use in fingerprinting of microbial communities by PCR-denaturing gradient gel electrophoresis. Appl. Environ. Microbiol. 70, 4800–4806. doi: 10.1128/AEM.70.8.4800-4806.2004 15294817 PMC492348

[B133] Zaidi A. Ahmad E. Khan M. S. Saif S. Rizvi A. (2015). Role of plant growth promoting rhizobacteria in sustainable production of vegetables: Current perspective. Scientia Hortic. 193, 231–239. doi: 10.1016/j.scienta.2015.07.020

[B134] Zhang Z. Qu Y. Li S. Feng K. Wang S. Cai W.. (2017). Soil bacterial quantification approaches coupling with relative abundances reflecting the changes of taxa. Sci. Rep. 7, 4837. doi: 10.1038/s41598-017-05260-w 28684789 PMC5500469

[B135] Zhao X. Zhao C. Niu Y. Choa W. He W. Wang Y.. (2022). Understanding and comprehensive evaluation of cold resistance in the seedlings of multiple maize genotypes. Plants 11, 1881. doi: 10.3390/plants11141881 35890515 PMC9320912

[B136] Zhu S. Vivanco J. M. Manter D. K. (2016). Nitrogen fertilizer rate affects root exudation, the rhizosphere microbiome and nitrogen-use-efficiency of maize. Appl. Soil Ecol. 107, 324–333. doi: 10.1016/j.apsoil.2016.07.009

