# Supplementary material for: Agronomic strategies to enhance the early vigor and yield of maize. Part I: the role of seed applied biostimulant, hybrid and starter fertilization on rhizosphere bacteria profile and diversity
Source: Front Plant Sci. 2023 Nov 2;14:1240310. doi: 10.3389/fpls.2023.1240310 (PMC10651756; doi:10.3389/fpls.2023.1240310)
Supplement: Supplementary file 1 [file Table_1.docx]

Supplementary Material

Agronomic Strategies to Enhance the Early Vigor and Yield of Maize. Part I: the Role of Seed Applied Biostimulant, Hybrid and Starter Fertilization on Rhizosphere Bacteria Profile and Diversity

Gergely Ujvári^1^, Luca Capo^2^, Arianna Grassi^1^, Caterina Cristani^1^, Irene Pagliarani^1^, Alessandra Turrini^1^, Massimo Blandino^2*^, Manuela Giovannetti^1^, Monica Agnolucci^1*^

^1^Department of Agriculture, Food and Environment, University of Pisa, Pisa, Italy

^2^Department of Agriculture, Forest and Food Sciences, University of Turin, Grugliasco, Italy

***Correspondence:**Monica Agnolucci

[monica.agnolucci@unipi.it](mailto:monica.agnolucci@unipi.it)

Massimo Blandino

[massimo.blandino@unito.it](mailto:massimo.blandino@unito.it)

# Supplementary Tables

**Table S1**. The main physical and chemical characteristics of the natural topsoil (0-30 cm) used in the growth chamber experiment.

| **Parameters** |  |
| --- | --- |
| Soil type | Typic Ustifluvent |
| Soil texture | Silt loam |
| Sand (0.05 - 2 mm) | 272 g kg^-1^ |
| Silt (0.002 - 0.05 mm) | 680 g kg^-1^ |
| Clay (< 0.002 mm) | 48 g kg^-1^ |
| pH (H_2_0) | 7.9 |
| Total carbonate (CaCO_3_) | 12 g kg^-1^ |
| Organic matter | 18.2 g kg^-1^ |
| C/N | 8.6 |
| Cation Exchange Capacity (C.E.C.) | 11.0 cmol(+) kg^-1^ |
| Total Nitrogen | 1.23 g kg^-1^ |
| Exchangeable Potassium | 52 mg kg^-1^ |
| Olsen Phosphorus | 6 mg kg^-1^ |

**Table S2.** The main agronomic information pertaining to the maize growing cycle in the growth chamber experiment. GDDs: accumulated growing degree days on a 10°C basis. PAR: photosynthetically active radiation: μmol m^-2^ s^-1^.

| Medium temperature of the air | Day | 16.7 | C° |
| --- | --- | --- | --- |
|  | Night | 14.1 | C° |
| Air GDDs | Sowing - 6 leaves | 273 | C°-day |
| Medium temperature of the soil | Day | 15.6 | C° |
|  | Night | 14.2 | C° |
| Soil GDDs | Sowing - 6 leaves | 237 | C°-day |
| Air Humidity | | 50 | % |
| Day/night | | 12 | h |
| Light intensity | | 700-1000 | PAR |
| Water irrigation | | 10 mm every 7 day | |

**Table S3.** Best match identification of bacterial sequences retrieved from PCR-DGGE analysis of the rhizosphere samples at emergence (**A**) and at 5 leaf-stage (**B**).

**A)**

| **Fragment** | **Taxon name** | **Isolation source** | **Closest match (% similarity)** | **GenBank accession number** |
| --- | --- | --- | --- | --- |
| **1** | *Pedobacter panaciterrae* CsMH-334 | karst caves | 100.00 | MT415191.1 |
| **2** | *Flavobacterium* sp. H86 | *Astragalus mongholicus* root endosphere | 99.24 | MN954285.1 |
| **3** | *Pedobacter steynii* TRB148 | soil | 99.43 | KX981359.1 |
| **4** | *Pedobacter* sp. CCBAU 10902 | maize rhizosphere | 99.42 | JF772566.1 |
| **5** | *Massilia eurypsychrophila* PWB9 | meltwater | 99.81 | ON420933.1 |
| **6** | *Massilia aurea* AP13/*Massilia atriviolacea* SOD | drinking water/soil | 98.12 | NR_042502.1/NR_171529.1 |
| **7** | *Paenibacillus* sp. ICMP 16203 | *Clianthus puniceus* root nodules | 98.25 | MK382483.1 |
| **8** | *Adhaeribacter terrae* HY02 | mountain soil | 98.68 | NR_157726.1 |
| **9** | *Pedobacter* sp. NT 4-05 | root endosphere | 99.25 | KM253140.1 |
| **10** | *Adhaeribacter terrae* HY02 | mountain soil | 97.30 | NR_157726.1 |
| **11** | *Janthinobacterium* sp. J1/ *Janthinobacterium lividum* Pie_T20 | *Medicago polymorpha* root endosphere/ *Typha latifolia* rhizoplane | 100.00 | MK007391.1/MG687520.1 |
| **12** | *Lysobacter* sp. RHLT3-4 | glaciers | 99.81 | JX949389.1 |
| **13** | *Lysobacter* sp. RB72 | spring water | 99.06 | FJ898300.1 |
| **14** | *Stenotrophomonas* sp. So3Pt_86 | forest soil | 99.44 | AB836481.1 |
| **15** | *Stenotrophomonas* sp. LpB5d | *Lotus parviflorus* root nodules | 99.41 | MT071934.1 |
| **16** | *Stenotrophomonas* sp. PN3-B04P1-9 | wheat rhizosphere | 99.81 | MK638446.1 |
| **17** | *Stenotrophomonas rhizophila* B1 | *Brassica* spp. seed endosphere | 100.00 | MN629046.1 |

**B**)

| **Fragment** | **Taxon name** | **Isolation source** | **Closest match (% similarity)** | **GenBank accession number** |
| --- | --- | --- | --- | --- |
| **18** | *Flavobacterium* sp. H86 | *Astragalus mongholicus* root endosphere | 99.42 | MN954285.1 |
| **19** | *Flavobacterium saccharophilum* PDW1006/*Flavobacterium collinsii* 0A03 | poultry drinking water/tundra soil | 98.46 | MZ642623.1/MH929886.1 |
| **20** | *Flavobacterium* sp. H86 | *Astragalus mongholicus* root endosphere | 99.04 | MN954285.1 |
| **21** | *Adhaeribacter terrae* HY02 | mountain soil | 97.88 | NR_157726.1 |
| **22** | *Pedobacter* sp. NT 4-05 | root endosphere | 97.40 | KM253140.1 |
| **23** | *Chryseolinea* sp. Jin1 | unknown | 95.72 | MT893350.1 |
| **24** | *Pedobacter* sp. NT 4-05 | root endosphere | 99.07 | KM253140.1 |
| **25** | *Limnobacter thiooxidans* H01Y-133 | *Prymnesium saltans* algal culture | 99.81 | MK493573.1 |
| **26** | *Lysobacter* sp. RHLT3-4 | glaciers | 98.17 | JX949389.1 |
| **27** | *Polaromonas ginsengisoli* Gsoil 115 | ginseng field soil | 99.24 | AB245355.1 |
| **28** | *Polaromonas ginsengisoli* Gsoil 115 | ginseng field soil | 99.43 | AB245355.1 |
| **29** | *Lysobacter* sp. YC6725 | rice field soil | 97.73 | EU707563.1 |
| **30** | *Stenotrophomonas rhizophila* B24 | *Brassica* spp. seed endosphere | 99.26 | MN629066.1 |
| **31** | *Stenotrophomonas* sp. SH-1.1-R-5 | *Dendrobium officinale* endosphere | 100.00 | MN784189.1 |
| **32** | *Stenotrophomonas* sp. LpB5d | *Lotus parviflorus* root nodules | 99.81 | MT071934.1 |
| **33** | *Stenotrophomonas* sp. T2BM2-2 | agave plant microbiota | 99.81 | OP210260.1 |
| **34** | Uncultured proteobacterium clone 39 | freshwater | 89.85 | MN844051.1 |
| **35** | *Lysobacter* sp. YC6725 | rice field soil | 97.93 | EU707563.1 |
| **36** | *Flavobacterium* sp. H86 | *Astragalus mongholicus* root endosphere | 99.42 | MN954285.1 |
| **37** | *Pedobacter panaciterrae* WR144 | ginseng rhizosphere soil | 100.00 | AB365796.1 |
| **38** | *Pedobacter panaciterrae* CsMH-334 | karst caves | 100.00 | MT415191.1 |
| **39** | *Pedobacter panaciterrae* CsMH-334 | karst caves | 99.81 | MT415191.1 |
| **40** | *Pedobacter steynii* TRB148 | soil | 100.00 | KX981359.1 |
| **41** | *Pedobacter* sp. NT 4-05 | root endosphere | 100.00 | KM253140.1 |
| **42** | *Pedobacter* sp. NT 4-05 | root endosphere | 99.81 | KM253140.1 |
| **43** | *Chryseolinea* sp. Jin1 | unknown | 96.33 | MT893350.1 |
| **44** | *Limnobacter thiooxidans* H01Y-133 | *Prymnesium saltans* algal culture | 99.81 | MK493573.1 |
| **45** | *Limnobacter thiooxidans* H01Y-133 | *Prymnesium saltans* algal culture | 99.62 | MK493573.1 |
| **46** | *Polaromonas ginsengisoli* Gsoil 115 | ginseng field soil | 99.63 | AB245355.1 |
| **47** | *Lysobacter concretionis* N3 | nickel sludge waste | 98.85 | MG788290.1 |
| **48** | *Lysobacter ginsengisoli* Gsoil 357 | ginseng field soil | 98.86 | NR_112563.1 |
| **49** | *Lysobacter* sp. YC6725 | rice field soil | 97.56 | EU707563.1 |
| **50** | *Stenotrophomonas* sp. 12C_21 | lake water | 100.00 | AY689084.1 |
| **51** | *Stenotrophomonas* sp. BIS1040 | soil | 99.43 | MN810222.1 |
| **52** | *Stenotrophomonas* sp. LpB5d | *Lotus parviflorus* root nodules | 99.81 | MT071934.1 |
| **53** | *Rhodanobacter* sp. movR-3 | rhizosphere soil | 99.81 | KY753356.1 |
| **54** | *Stenotrophomonas* sp. V10R15 | *Phragmites australis* roots | 99.25 | MT165571.1 |
| **55** | *Stenotrophomonas* sp. V10R15 | *Phragmites australis* roots | 98.66 | MT165571.1 |
